# Spatial Designs on Metamaterial Sensors for Enhancing Signals and Detecting Extracellular Vesicles

**DOI:** 10.1021/acsami.5c07175

**Published:** 2025-10-16

**Authors:** Esma Derin, Eylul Gulsen Yilmaz, Özgecan Erdem, Yusuf Aslan, Abdullah Kafadenk, Süleyman Çelik, Ümit Çelik, Fatih Inci

**Affiliations:** 1 UNAM − National Nanotechnology Research Center, Bilkent University, Cankaya, Ankara 06800, Turkey; 2 Institute of Materials Science and Nanotechnology, Bilkent University, Cankaya, Ankara 06800, Turkey; 3 Sabanci University Nanotechnology and Application Center (SUNUM), Sabanci University, Orhanli, Tuzla, Istanbul 34956, Turkey; 4 School of Civil Aviation, Firat University, Merkez, Elazig 23119, Turkey

**Keywords:** metamaterial sensors, optical disks, nanoislands, extracellular vesicles, signal enhancement

## Abstract

Biosensors, while holding immense promise for biomarker detection, face substantial challenges in analytical performance, fabrication intricacies, and complex applications, hindering their seamless integration into point-of-care (POC) settings. Metamaterial-based plasmonic biosensors offer tremendous potential for biomarker detection; however, their widespread adoption in POC diagnostics remains hampered by limitations in sensitivity, fabrication complexity, and production cost. Herein, we introduce, for the first time, in situ-controlled spatial designs on metamaterial-based plasmonic sensors, demonstrating unprecedented sensitivity in detecting extracellular vesicles (EVs). In the fabrication process, commercially available optical disks were repurposed as nanostructured substrates, yielding a cost reduction of up to 260-fold ($0.90 per sensor) and a fabrication time reduction of approximately 960-fold, compared to conventional e-beam lithography. Leveraging inherent nanogratings, measurements are conducted on a compact, palm-sized platform, addressing challenges in usability and portability associated with bulky optical designs. Through ex situ immobilization of gold nanoparticles (AuNPs) or in situ formation of nanoislands (NIs), we have engineered plasmonic hotspots that substantially enhanced local electric field intensities, thereby amplifying the bulk refractive index sensitivity of the sensors. Finite-difference time-domain simulations confirmed that the spatial arrangement and interparticle distances of spatial designs enhance near-field effects. The optimized platform exhibits up to a 5.5-fold enhancement in refractive index sensitivity. Moreover, based on data obtained from nanoparticle tracking analysis (NTA), fluorescence-enhanced NTA (fNTA), and recent literature benchmarks, the platform demonstrated detection limits of 10^4^ particles/μL (as determined by raw NTA measurements), approximately 330 fg/μL (estimated via literature-based EV mass calculations), and 138 EVs/μL (quantified via fNTA using marker-specific labeling). Herein, we anticipate that repurposing disks as metamaterial sensors has the potential to address pressing challenges in usability, portability, cost, and complexity. Besides, 3D configurations on sensors would improve the analytical performance, offering highly sensitive and facile platforms for diverse applications in the future.

## Graphical abstract

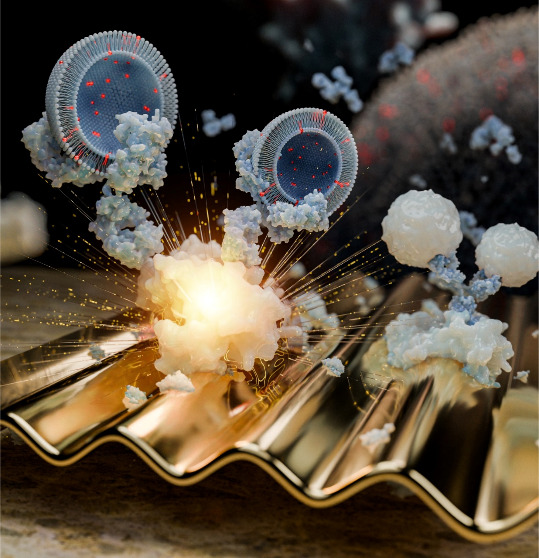

## 1 Introduction

Today, biosensors are facilitated to detect distinct biomarkers in the settings of hospitals, clinics, and research laboratories.^1–5^ Basically, intramolecular interactions and molecule-material interfaces are tracked through a myriad of detection modalities such as electrical,^6^ mechanical,^7^ and optical sensing schemes.^8^ Optical sensors, in particular, measure the remote transduction of biomolecular binding within a confined volume while they do not have any physical connections with the excitation source.^9,10^ They also enable understanding biomolecular interactions directly through the decorated chemistry on the sensors.^11^ Optical modality is also compatible with biological matrices—despite having interfering factors such as ionic contents. However, this scheme mostly requires a well-alignment between a coupling unit (e.g., a prism, grating, and waveguide structures) and biological interface^12^ that remarkably hampers their expansions into the point-of-care (POC) settings.

Metamaterials are artificial electromagnetic media^13^ that exhibit superior optical assets, and they can be fabricated as periodic or special arrangements of metal/dielectric elements at subwavelength dimensions that manipulate light through spatially arranged meta-atoms.^14,15^ Metamaterials with plasmonic assets have allowed improving analytical performance in biosensing approaches, and at the same time, they have minimized the need for complex designs of optics.^16^ Despite the mutuality of metamaterials and plasmonics, high-cost, lengthy, and complex fabrication procedures cannot still resolve the challenges for their adaptation into the POC settings.^17^ In this context, modifying inherently existing nanostructures on optical disks has been introduced as an alternative strategy to tackle some of the aforementioned challenges in their fabrication.^16,18–20^ Although the specific body of proof-of-concept work and minimal assessments in analytical performance exist in the literature,^21^ this strategy was able to provide small perturbations at the resonance wavelength upon the binding of nanosized particles. Therefore, low sensitivity still remains an analytical challenge. In particular, in order to improve the sensitivity, spatial designs are integrated with metamaterial-based plasmonic sensors.^22^ Conventional lithographic methods are however intricate, time-consuming, high-cost, and low-yield in production, and they are prone to interpersonal errors.^23^ However, the advent of diverse unconventional nanofabrication methods has radically changed the production of subwavelength nanostructures. Among these methods, solid-state dewetting,^24^ self-assembly,^25^ and seed-mediated growth^26^ became prominent on large surface area and cost-effective fabrication of metal nanoislands (NIs). Depending on interparticle distance between NIs, myriad nanogaps formed between the NIs yield intense localized plasmonic hot spots.^23^ An increase in both the amount and the evanescent field intensity of these hotspots boosts the interaction of localized surface plasmons with their surroundings. In addition, the size, shape, and distribution of the NIs can be tuned with the change of fabrication parameters. Considering nanogap enrichment and nanostructure tunability, NIs benefit diverse nanophotonic and surface-sensitive applications, ranging from biosensors to tunable optoelectronic devices. Although constructing NIs and integrating metal nanoparticles are facile strategies to generate hot-spots through spatial confinements for boosting sensitivity, agglomeration/aggregation of nanoparticles and the use of harsh chemicals interfere with the periodic structure of sensors, leading to irreversible signal loss.^27^ Thus, *in situ* control of spatial configurations would hold great potential to fine-tune and amplify the signals for improving the sensitivity.

In this study, we introduce, for the first time, an innovative approach involving in situ controlled spatial designs on metamaterial-based plasmonic sensors specifically tailored for the detection of nanosized extracellular vesicles (EVs) from biologically relevant matrices (Figure 1a). The initial step involved repurposing optical disks with inherent nanograting structures for reducing fabrication costs significantly. Subsequently, the top surface of optical disks was coated with plasmonic alloys (gold, silver, and titanium; referred to as gold-top) (Figure 1b) or silver (only silver coating; referred to as silver-top) (Figure 1e), followed by the application of poly-l-lysine (PLL) as a dielectric adlayer. Integration of ex situ produced gold nanoparticles (AuNPs) (Figure 1c,f) and in situ formation of NI (Figure 1d,g) on these sensors led to the assessment of novel spatial configurations, evaluating signal enhancements for both bulk and binding sensitivity in EV detection. The cross-sectional electric field profiles (Figure 1b–g) and reflection spectra of these metamaterial sensors were rigorously studied by using the finite difference time domain (FDTD) method. Furthermore, this spatial metamorphosis was examined for the binding sensitivity by comparing data with their bare counterparts while detecting EVs from physiological buffers, artificial urine, and plasma. All sensing operations were simply computed using a hand-held device operated with our user-friendly, in-house software. From the perspectives of EV research and metamaterials, the manifold achievements detailed below underscore the significant contributions of this research in terms of novelty, enhanced sensitivity, reduced fabrication cost, and assay time. Consequently, we anticipate that our spatial design strategy would resolve the prevailing cost- and complex fabrication-related challenges in metamaterial-based sensors, offering an affordable and facile diagnostic platform for the biosensing realm.

**Figure 1:**
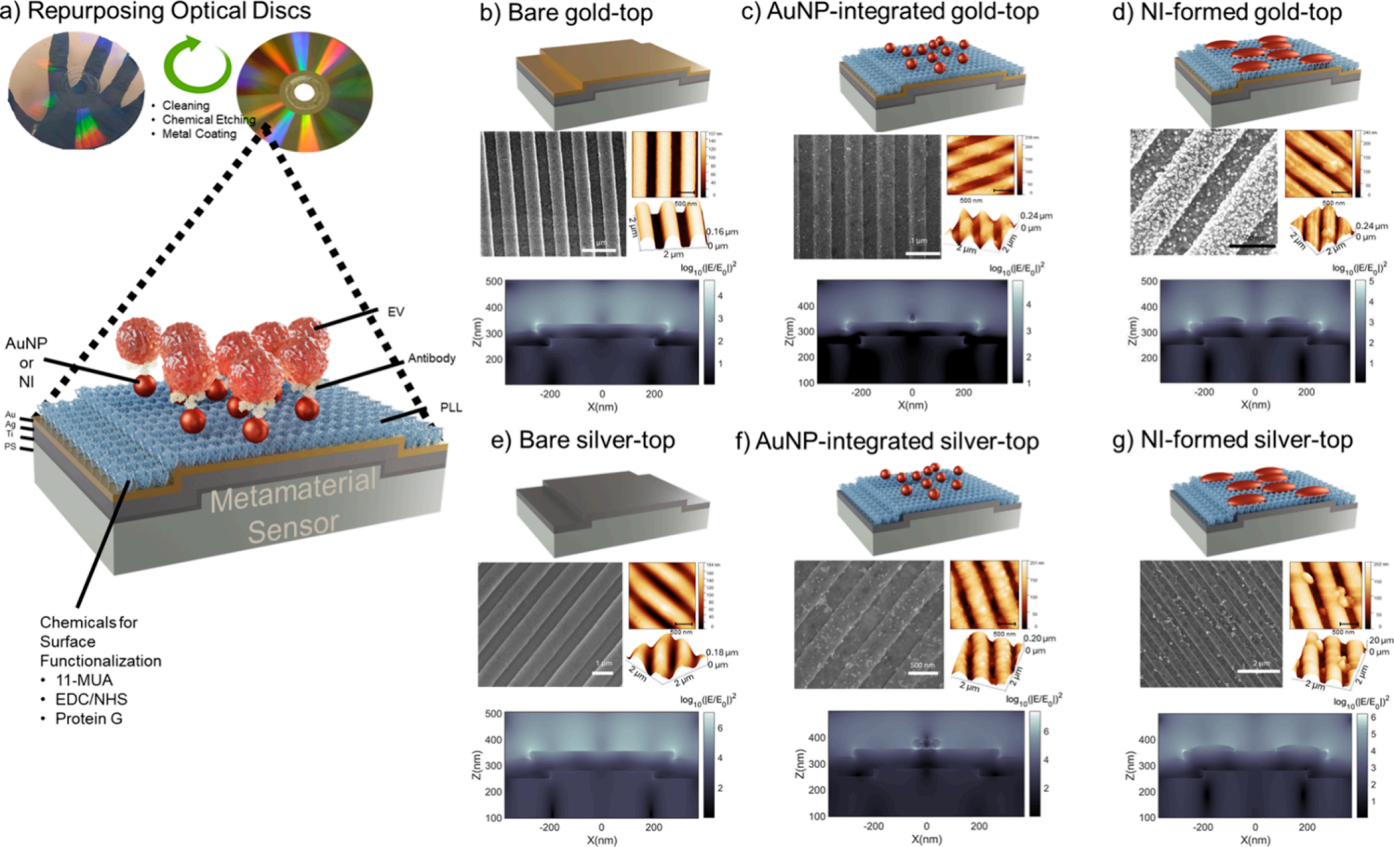
Schematic and characterization of repurposed optical disk-based metamaterial sensors. (a) Optical disks are repurposed as gold- and silver-top metamaterial plasmonic sensors, further modified via either ex situ integration of AuNPs or in situ formation of NIs for EV detection. Topographical characterization using SEM and AFM is presented for the (b) bare gold-top sensor, (c) AuNP-integrated gold-top sensor, (d) NI-formed gold-top sensor, (e) bare silver-top sensor, (f) AuNP-integrated silver-top sensor, and (g) NI-formed silver-top sensor. Corresponding FDTD simulations for each surface morphology illustrate the electric field distribution at resonance wavelengths for each sensor configuration.

## 2 Results and Discussion

### 2.1 Repurposing Disks as Metamaterial Sensors and Characterization Studies

#### 2.1.1 Optical Disk Substrate Preparation and Surface Engineering

In this study, we repurposed commercially available optical digital versatile disks (DVDs) as metamaterial sensor substrates by leveraging their intrinsic nanograting structures, which feature continuous periodicity over large surface areas (Figure 1a). This inherent nanostructure allowed us to utilize the plastic substrate (PS) of the optical disks as the foundation for metamaterial sensor fabrication, circumventing the need for complex and costly lithographic techniques typically required to achieve nanoscale periodicity. The optical disks were first cleaned using physical methods and subsequently etched chemically to further refine the nanograting structure, following established protocols.^14,28^

#### 2.1.2 Surface Topography Analysis of Bare and Modified Sensors

Surface topography of the sensor platforms—including bare, AuNP-integrated, and NI-formed surfaces—for both gold- and silver-top configurations was characterized via scanning electron microscope (SEM) and atomic force microscope (AFM) (Figure 1b–g). Assessing the SEM images (Figure 1b,e), bare gold- and silver-top sensors had smooth surfaces and edges. As is clearly visible in Figure 1c,f, AuNP integration occurred similarly for both gold- and silver-top sensor surfaces. In addition, we observed that NI structures were homogeneous and largely distributed over the periodic structures of the gold-top metamaterial sensor compared to the NI distribution on the silver-top metamaterial sensor (Figure 1d,g). Corresponding height profiles are presented in Figure S1, detailing the nanograting dimensions for each design. On the bare gold-top surfaces, the measured grating features were ∼400 nm in width and ∼270 nm for the spacers (Figure S1a). Similarly, silver-top surfaces exhibited an ∼380 nm width and ∼270 nm spacing (Figure S1d). Upon integration of a biomaterial adlayer, we visualized AuNP localization on both gold- and silver-top sensors with comparable distributions across the PLL-modified surfaces (Figure S1b,e). For AuNP-integrated gold-top sensors, nanograting parameters were ∼540 nm in width with ∼270 nm spacing (Figure S1b), while silver-top surfaces showed ∼620 nm width and ∼190 nm spacing (Figure S1e). After the biomaterial layer, NI localization was also observed on both gold- and silver-top sensors (Figure S1c,f). Although optical disks typically have a 740 nm periodicity, slightly reduced values were consistently recorded. This discrepancy (approximately 10%) may stem from the limited scanning area in AFM, tip geometry, or calibration-related variances.

#### 2.1.3 Characterization of AuNPs and Nanoislands

Additionally, ex situ synthesized AuNPs and in situ formed NIs were integrated to create spatial configurations on the sensors, and their characteristics were analyzed by dynamic light scattering (DLS) and transmission electron microscope (TEM) (Figure S2), where the diameters of ex situ produced AuNPs were initially measured as ∼35.59 and 25.59 ± 6.27 nm, respectively (Figure S2a–c). Since DLS measures a hydration layer around nanoparticles, around 10 nm higher diameter was observed in this method. When 200 μM seeding solution was introduced to the AuNP suspension, DLS revealed a size increase to 46.77 nm (Figure S2d), indicating ∼11 nm growth attributed to surface seeding. In a parallel experiment designed to simulate the in situ NI formation (without AuNPs), we measured the effect of seeding solution for understanding the in situ formation of NI in solution, and it was calculated as 47.30 nm (Figure S2e). Moreover, when the same experiment was performed with a higher concentration of seeding solution (250 μM), the size of the NI structures was observed as 49.34 nm (Figure S2f).

#### 2.1.4 In Situ Formation and Characterization of Nanoislands on Sensor Surfaces

In situ formation of NIs was achieved directly on the sensor surface by chemically reducing chloroauric acid (HAuCl_4_) with hydroxylamine hydrochloride (HONH_2_·HCl) on PLL-modified substrates.^29^ This method simultaneously facilitated both AuNP formation and surface seeding. In our study, the term “in situ control” denotes our capacity to modulate the formation of NIs directly on the sensor surface through the introduction of a seeding solution while simultaneously monitoring the optical signal in real-time. This strategy allows precise determination of optimal parameters—specifically, solution concentration and application duration—that yield the most pronounced plasmonic response without compromising the nanograting’s periodicity or disrupting the optical signal.

As shown in Figure 1d, the resulting NIs presented broad size distributions with characteristic hemi ellipsoidal morphologies (heights of 19.3 and 24 nm; lengths of 177 and 157 nm). Particle analysis using NIH ImageJ software indicated relatively uniform surface coverage (∼54%) with preferential localization on top surfaces rather than within nanograting grooves. Maintaining the integrity of the underlying nanostructure was essential for effective NI formation; overexposure to seeding solutions or extended reaction durations disrupted the periodicity and attenuated the plasmonic signal. In addition, gold-top sensors subjected to in situ NI formation exhibited rougher and more irregular surface morphologies, suggesting possible interactions between the gold layer and the chemicals used, which altered the periodic nanostructure. In contrast, in situ NI formation was sparse on the silver-top surface, mainly localizing the grooves of the nanoperiodic structures.

#### 2.1.5 Comparative Surface Morphology of Gold- and Silver-Top Sensors

After integrating a biomaterial adlayer, we imaged the decoration of NI structures on both gold-top and silver-top sensors and again observed similar localization patterns on the PLL-modified sensors (Figure S1c,f). Nanograting parameters for the NI-modified gold-top surfaces were ∼555 nm width and ∼280 nm spacing (Figure S1c), while silver-top counterparts showed ∼600 nm width and ∼200 nm spacing (Figure S1f).

#### 2.1.6 Surface Chemistry Characterization by XPS

To assess surface chemistry and verify the presence of elemental components after each modification step, X-ray photoelectron spectroscopy (XPS; K-Alpha, ThermoFisher Scientific, USA) was performed (Figure S3). XPS analysis quantified the elemental composition and chemical states via the photoelectric effect. On gold-top sensors, Au 4f and N 1s spectra were acquired for bare, PLL-modified, AuNP-integrated, and NI-formed surfaces. For silver-top sensors, Ag 3d spectra were also recorded alongside Au 4f and N 1s signals under the same conditions.

### 2.2 Modeling of Metamaterial Sensors

The optical behavior of metamaterial sensors functionalized with AuNPs and NIs was systematically investigated by using two-dimensional FDTD simulations in Lumerical. These simulations, based on experimentally determined structural parameters, enabled the evaluation of both the absorbance spectra and the spatial distribution of local electric field intensities, thereby providing detailed insight into the optical behavior of sensor configurations incorporating AuNPs and NIs.

#### 2.2.1 Topographical Characterization and Simulation of Gold-Top Sensors

Initial characterization of the gold-top metamaterial sensors was performed by using AFM (Figure 2a). Following ex situ immobilization of AuNPs onto the biomaterial adlayer, a sparse distribution of particles was observed along the nanograting structure. Height profiles confirmed the AuNP diameter (*D*) to be approximately 30 nm, consistent with DLS and TEM analyses (Figure S2). Subsequently, the computational unit cell representing the integration of AuNPs onto the gold-top metamaterial sensor was devised featuring a singular AuNP centrally positioned within the nanograting structure (Figure 2b). The AuNP diameter was iteratively tuned in the model to match the normalized experimental absorption spectrum, with the optimal agreement achieved at a diameter of 24 nm (Figure 2c). This dimension was in line with both the TEM and DLS measurements, validating the simulation geometry. Subsequent topographic analysis of the NIs formed in situ on gold-top sensors revealed a single NI with a height of ∼19.3 nm and a length of ∼177 nm (Figure 2d). Further scrutiny of the NIs on the gold-top metamaterial sensor is presented in Figure S4. Subsequent analysis of the same NI revealed measurements of 24 nm for the height and 157 nm for the length. Examination from varied perspectives indicated a geometric configuration closely resembling that of a hemi ellipsoid. Since the simulations were reliant on 2D geometry, the hemi ellipsoid shape was converted to a hemi ellipse. Consequently, based on the cross-sectional profile of the hemi ellipsoid, the radii values of the corresponding hemi ellipse were determined to be 24 and 85 nm along the X- and *Y*-axes, respectively (Figure 2e). The attainment of the normalized absorption spectrum for the modeled hemi ellipses on the gold-top metamaterial sensor was facilitated by adjusting the interparticle distance (*L*) between the hemi ellipses. The optimized normalized spectrum was consistent with the experimentally acquired normalized absorption spectrum of the AuNP-integrated gold top metamaterial sensor when the *L* value was equal to 122 nm (Figure 2f).

**Figure 2:**
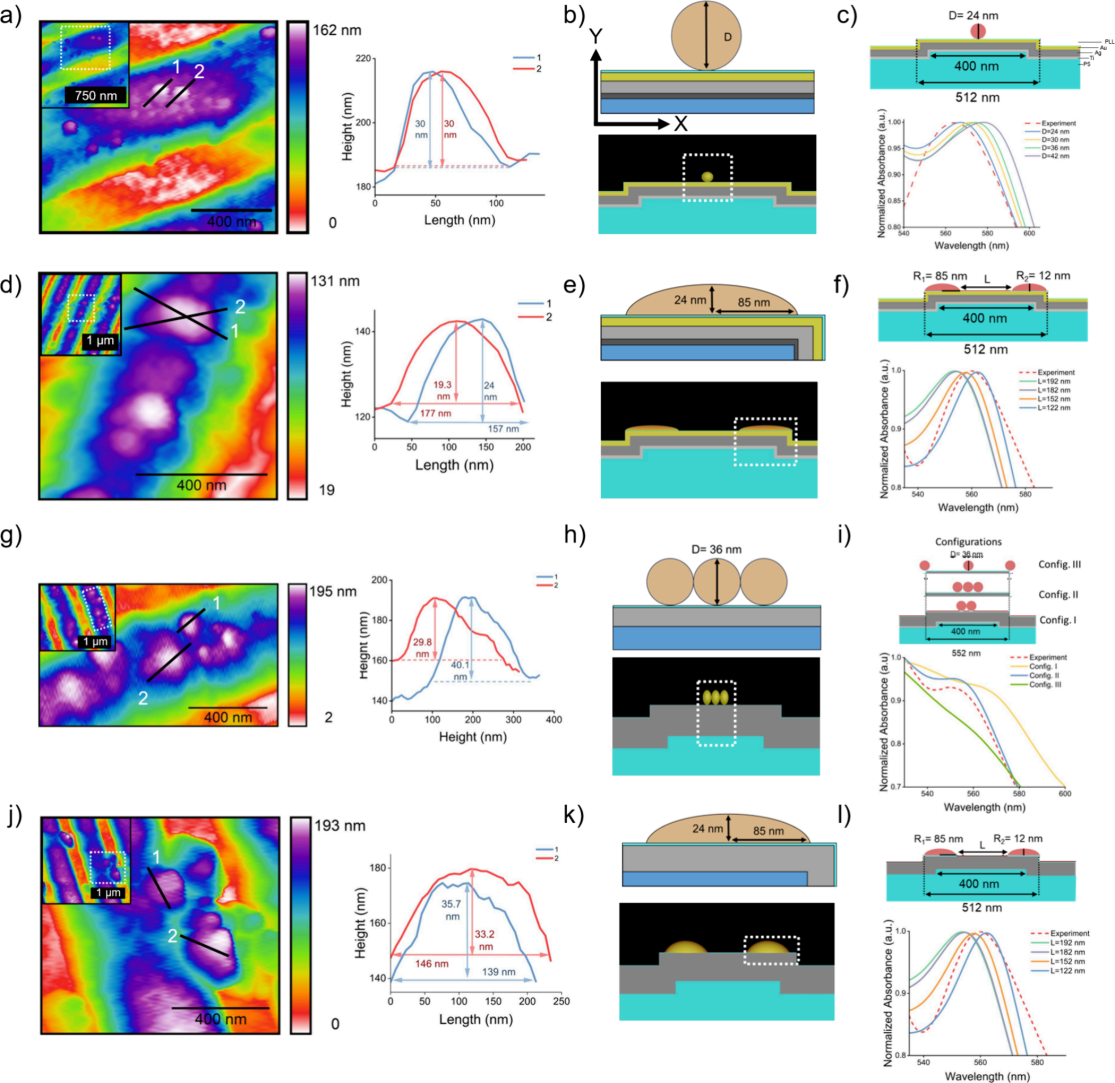
FDTD modeling of NIs and AuNPs on gold- and silver-top metamaterial sensors based on experimentally acquired topographies. (a) Large-scale and zoomed AFM maps of the AuNP-integrated gold-top sensor. (b) FDTD simulation geometries incorporating these structural parameters are shown for the AuNP-integrated gold-top sensor. (c) Simulated normalized absorbance spectra as a function of AuNP diameter on gold-top sensors. (d) Large-scale and zoomed AFM maps of the NI-formed gold-top sensor. (e) FDTD simulation geometries incorporating these structural parameters are shown for the NI-formed gold-top sensor. (f) Simulated changes in normalized absorbance spectra with varying interparticle distances between NIs on the gold-top sensor. (g) Large-scale and zoomed AFM maps are shown for the AuNP-integrated silver-top sensor. (h) FDTD simulation geometries incorporating these structural parameters are shown for the AuNP-integrated silver-top sensor. (i) Simulated changes in normalized absorbance spectra with varying configurations of AuNPs on the silver-top sensor. For silver-top sensors, different AuNP configurations were modeled to extract their spectral response. (j) Large-scale and zoomed AFM maps are shown for NI-formed silver-top sensors. (k) FDTD simulation geometries incorporating these structural parameters are shown for the NI-formed silver-top sensor. Simulated changes in normalized absorbance spectra with varying interparticle distances between NIs on the silver-top sensor. FDTD simulation geometries incorporating these structural parameters are shown with annotated particle dimensions and unit-cell simulation regions indicated below each model.

#### 2.2.2 Topographical Characterization and Simulation of Silver-Top Sensors

Parallel modeling and characterization procedures were applied to the silver-top sensors. AFM analysis revealed a denser AuNP distribution compared to gold-top counterparts (Figure 2g), with measured diameters of 29.8 and 40.1 nm, again in agreement with DLS and TEM data. To reflect this denser population, simulation unit cells were constructed by using multiple AuNP configurations (Figure 2h). The normalized absorption spectra of these configurations were juxtaposed with the experimentally obtained data from the AuNP-incorporated gold-top sensor, and the configurations for AuNP adsorption were simulated accordingly (Figure 2i). The optimized unit cell featured three sequential AuNPs positioned at the center of the nanograting structure. Subsequently, NI formation was examined on silver-top sensors similar to that of the NI-formed gold-top counterparts. Examination of the topography revealed two distinct NIs on the silver-top metamaterial sensor, displaying radius values of 35.7 and 33.2 nm along the *Y*-axis and 139 and 146 nm along the *X*-axis, respectively (Figure 2j). Despite slight variations in the dimensions of the ellipsoidal profiles compared to those observed on the gold-top sensor, identical radius values were applied to configure the simulation unit cell for the NI-formed silver-top sensors (Figure 2k). The normalized absorbance spectrum of the modeled NI-formed silver-top sensors was simulated with different *L* values, similar to the NI-formed gold-top sensors (Figure 2l). The spectrum was fine-tuned to closely match the experimentally derived normalized absorption spectrum of the NI-formed silver-top sensor, ultimately converging when the *L* value reached 152 nm (Figure 2l). A comprehensive comparison between the simulation and experimentally acquired normalized absorption spectra of the sensors is succinctly summarized in Figure S5.

#### 2.2.3 Electric Field Distribution and Hot-Spot Formation

FDTD simulations also provided insight into the spatial distribution of the local electric fields. In unmodified gold- and silver-top metamaterial sensors, hot-spots naturally emerged at the nanograting corners as demonstrated in Figure 1b,e. Upon AuNP (Figure 1c,f) and NI (Figure 1d,g) integration, new hot-spot locations appeared.

In the AuNP-integrated sensors, SEM and AFM imaging confirmed a uniform AuNP distribution across the nanograting, with an average diameter of ∼30 nm. These particles introduced additional surface nanogaps and enhanced surface roughness, which modified the periodic morphology—measured at ∼540 nm period and ∼270 nm depth for gold-top sensors and ∼620 nm period and ∼190 nm depth for silver-top surfaces (Figure S1e,b). These features significantly influenced the local electric field distribution.

Simulations demonstrated that AuNPs created localized nanogaps at the AuNP–substrate interface, functioning as plasmonic hot-spots with enhanced near-field intensities compared with bare nanogratings. These findings were consistent with experimentally observed red-shifts and broadened absorption spectra, confirming a substantial amplification of the plasmonic response. Among all sensor configurations, the AuNP-integrated silver-top sensor exhibited the highest field enhancement (Figure 1f). This performance is attributed to synergistic plasmonic coupling, both among adjacent AuNPs and between the AuNPs and the underlying silver metamaterial surface, culminating in a superior plasmonic platform for high-sensitivity sensing applications.

For NI-formed sensors, SEM and AFM confirmed well-dispersed structures with an average lateral size of ∼167 nm on gold-top surfaces. These structures induced additional nanogaps and roughness, further enhancing the electric field localization. The improved bulk sensitivity observed in the NI-modified sensors is attributed to the synergistic plasmonic coupling effect. Importantly, the in situ formation strategy affords superior spatial control and alignment with the underlying nanograting, resulting in more stable and sharper plasmonic resonance peaks.

In summary, the strategic incorporation of AuNPs and NIs into metamaterial sensors introduces additional localized plasmonic regions, or hot-spots, primarily located at nanogaps between metallic features and metal–dielectric interfaces. These hot-spots significantly concentrate local electric fields, thereby increasing the sensor’s sensitivity to analyte interactions and local refractive index variations. As a result, resonance wavelength shifts become more pronounced and distinct. The combined insights from experimental characterization and FDTD simulations confirm that precise modulation of surface morphology and spatial distribution significantly amplifies the plasmonic performance, thereby advancing the design of highly tunable and practical sensing platforms.

### 2.3 Benchmarking the Bulk Sensitivity of Metamaterial Sensors

While assessing the analytical performance of the sensor, bulk sensitivity with portable measurement platform was initially evaluated (Figure 3). The optical path, 3D design, and actual photograph of the portable measurement platform are presented in Figure S6a–d. The microfluidic channel components and their assemblies with a sensor are demonstrated in Figure S6e,f. Using this setup, the microfluidic-integrated metamaterial sensors (silver-top, gold-top, and their modified variants) were tested with a series of glycerol solutions ranging from 0% (only distilled water) to 70% in order to evaluate their bulk refractive index sensitivity under controlled conditions (Figure 3a). A linear correlation was exhibited in terms of resonance wavelength shifts and refractive index changes (Figure 3b). The corresponding refractive index values (1.333–1.428) were incorporated into the FDTD simulations to investigate the optical response under controlled conditions, yielding a trend consistent with the experimental observations (Figure 3c).

**Figure 3:**
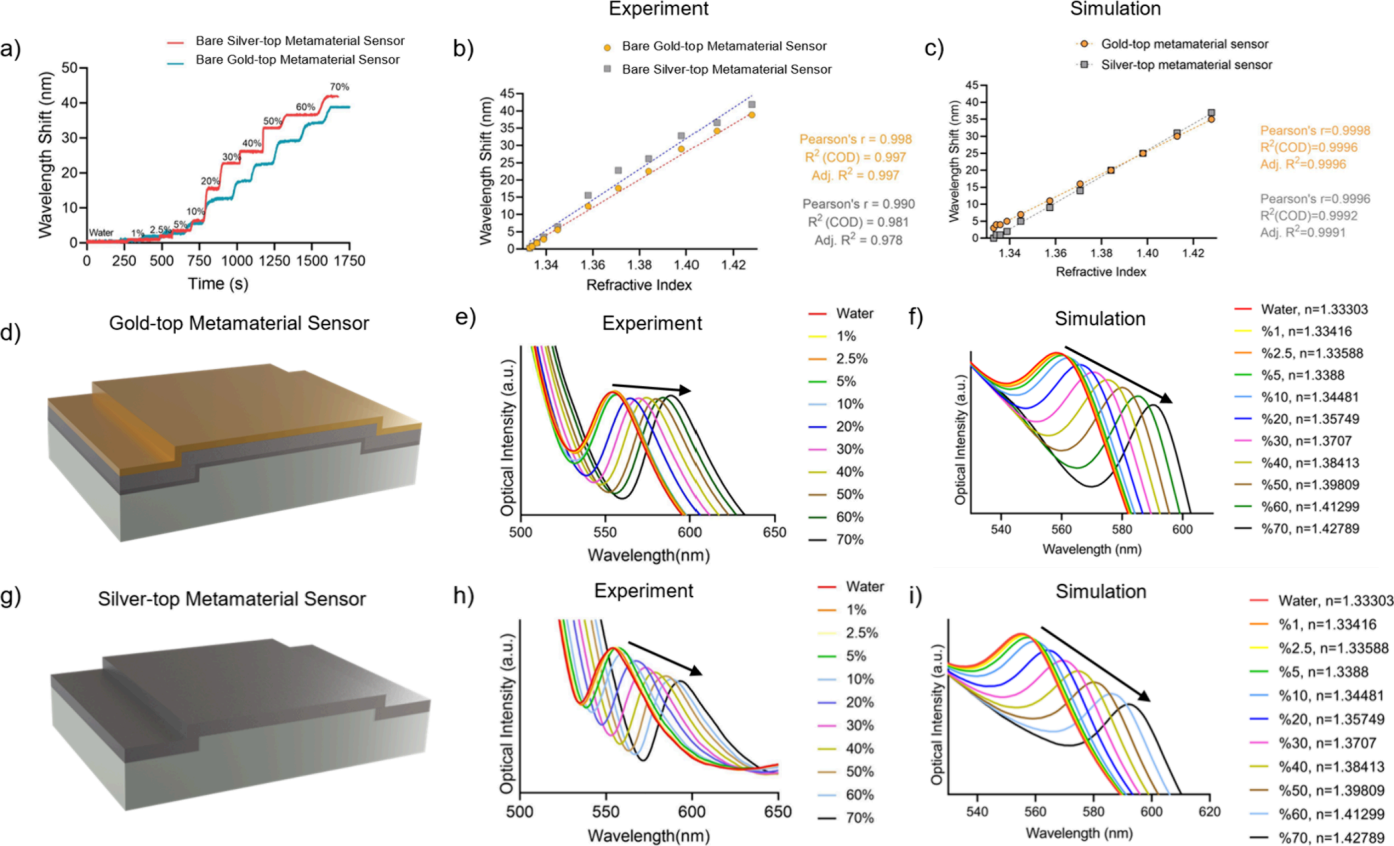
Benchmarking the bulk refractive index sensitivity of bare gold- and silver-top sensors using glycerol solutions. (a) Plasmonic resonance shifts observed on gold- and silver-top sensors across varying glycerol concentrations (1–70%). (b) A linear correlation is established between the refractive index values and resonance wavelength shifts for both sensor types. (c) The corresponding refractive index values (1.333–1.428) derived from glycerol concentrations were used in FDTD simulations for gold-top (d) and silver-top (g) sensors, also demonstrating a linear optical response. (e–f) Experimentally measured resonance shifts and corresponding simulated results for gold-top sensors, and similarly, (h–i) for silver-top sensors, further validate the bulk sensitivity performance of the metamaterial designs.

While assessing the entire spectra in the measurements, the sensors responded in red-shifts proportional to distinct glycerol solutions in terms of both dip and peak resonances (Figure 3d–i). For instance, while passing distilled water, the bare gold-top sensors provided a dip resonance at 531.25 nm and a peak resonance at 554.47 nm (Figure 3d–e), whereas the bare silver-top sensors had a dip resonance at 534.75 nm and a peak resonance at 554.17 nm (Figure 3g–h). While applying 70% of glycerol, these dip and peak resonances further red-shifted to 560.19 and 588.88 nm for the gold-top sensors and 571.70 and 592.91 nm for silver-top sensors. Likewise, the analytical performance was also simulated by changing the refractive index of the surrounding medium with corresponding refractive index units of glycerol concentrations (Figure 3f,i), and the simulation results showed the similar trend observed in the experiments. Further benchmarking these sensors, refractive index sensitivity (*S*) was calculated by dividing the magnitude of resonance shift to refractive index change during the measurements (eq 1).^30^ Accordingly, the silver-top metamaterial sensors were found to be more sensitive (438 nm/RIU) than the gold-top sensors (405.47 nm/RIU).

$$\mathrm{refractive}\;\mathrm{index}\;\mathrm{sensitivity}=\frac{\Delta \lambda }{\Delta n}$$ 1

As summarized in Figure S7, the impact of NI formation, AuNP integration, seeding concentration, and PLL surface modification on resonance wavelength shifts was systematically assessed. These evaluations were performed using varying concentrations of glycerol to benchmark the bulk refractive index sensitivity of the sensors. In order to evaluate the impact of surface chemistry on sensitivity, we first analyzed NI formation on the gold-top sensors. Briefly, fabricating NI structures typically includes conventional nanolithographic methods and dewetting.^23^ On the other hand, nanoparticles or NIs can be grown on the substrate or nanostructure through a chemical growth strategy, which relies on solution-based synthesis of nanoparticles such as AuNPs.^31^ The development of NI on a substrate is simply carried out by mixing ammonium hydroxide and chloroauric acid,^29,31^ and accordingly, their optical properties could be further tuned by changing seed density, shape of particles, or applied concentrations in the reaction.^31^ As a control, NI formation was performed on unmodified gold-top surfaces; however, no resonance shifts were observed under these conditions, pointing out the need for PLL as an adlayer for NI formation (Figure S7a). Hence, tuning the sensitivity would be possible to optimize the concentrations of both PLL and seeding solution. Accordingly, we observed the highest *S* value as 546.42 nm/RIU while modifying the sensors with 0.5 mg/mL of PLL along with 10 μM seeding solution. Moreover, the lowest *S* value (500.96 nm/RIU) was obtained from 1 mg/mL PLL and 10 μM seeding solution (Figure S7b), pointing out that the NI formation boosted the sensitivity between 123% and 135% with different NI formations compared to its bare counterparts. Following the optimization of PLL concentration, a series of seeding solution concentrations was evaluated to enhance NI formation efficiency (Figure S7c). Among the tested conditions, the addition of 10 μM seeding solution yielded the most favorable performance in terms of sensor response.

Instead of in situ NI formation, we also considered integrating AuNPs with PLL-modified sensors as the second possibility for signal enhancement due to their intrinsic plasmonic fashion. Once the PLL concentration (0.5 mg/mL) was determined, we hence evaluated the optimum conditions for the integration of AuNPs (∼30 nm) to the PLL-modified sensors. Initially, we diluted the stock AuNP solutions with 1:2, 1:5, 1:10, 1:20, and 1:50 ratios, and various glycerol solutions were applied to determine bulk sensitivity (Figure S7d). As a result, the *S* values were observed as 391.76 nm/RIU for a 1:5 dilution ratio, 496.84 nm/RIU for a 1:10 dilution ratio, 353.16 nm/RIU for a 1:20 dilution ratio, and 365.84 nm/RIU for a 1:50 dilution ratio. Overall, 1:10 dilution of the stock solution (0.200 OD (a.u.) at 526 nm of resonance) led to higher signal enhancements compared to the others and increased the signal by ∼130% compared to the bare version.

Considering all the results for the gold-top sensors, we realized that the concentrations of AuNPs and NIs were the mainstay for the enhancement; however, their high concentrations resulted in the loss of the resonance (Figure S8). For instance, a 100 μM seeding solution—prepared by mixing 200 μM HAuCl_4_ and 200 μM HONH_2_·HCl—was introduced into the microfluidic channel following baseline stabilization with distilled water (Figure S8a). Upon reaching the sensor surface, the seeding solution induced the immediate formation of NIs, as evidenced by a prompt plasmonic resonance wavelength shift. Notably, within approximately 2 min of solution flow at 10 μL/min, the resonance wavelength exhibited a peak red-shift of ∼10 nm, followed by a gradual blue-shift. Concurrently, as shown in Figure S8b, the resonance intensity declined progressively and was nearly extinguished after 5 min of exposure. These observations underscore the importance of finely tuning the seeding solution concentration and incubation duration to safeguard the nanograting structure and enable controllable in situ NI formation. A similar outcome was observed with an excessive amount of AuNP integration onto the surface, leading to a loss of resonance (Figure S8c).

We further assessed these modifications on the silver-top versions. In the case of AuNP integration, the *S* value increased to 515.75 nm/RIU, resulting in ∼118% of signal enhancement compared with the bare version (Figure S9a). While analyzing plasmonic signals in the NI fabrication, we observed that excessive numbers of particles led to a loss or decrease in the signal. On the silver-top sensors, the formation of NI structures was spatially confined and exhibited limited surface coverage, likely due to the intrinsic surface energy and lower chemical reactivity of the silver layer, which may have hindered uniform nucleation and growth. This limitation may also be attributed to the absence of gold nanostructures that, in gold-top sensors, facilitate and enhance particle initiation and growth during NI formation.

Considering their results in bulk sensitivity, the NI formation reduced the *S* value down to 1.12 times compared to the bare version (Figure S9b). Locally formed aggregates on the periodic structure might hinder the signals for silver-top sensors, which led to a decrease in bulk sensitivity. Per the calculations for the bulk sensitivity of each sensor, the highest enhancement was observed in AuNP-integrated gold-top sensors compared to that of bare gold-top counterparts.

### 2.4 Detecting Extracellular Vesicles

To evaluate the binding sensitivity of our plasmonic sensor platform, we employed EVs derived from human embryonic kidney 293 (HEK293) cells as a model biomarker. These EVs were selected based on emerging evidence highlighting their potential role in the early diagnosis of kidney diseases.^32,33^ The detection of HEK293-derived EVs served as a proof-of-concept, demonstrating the capability of the platform to identify nanoscale biological targets. Furthermore, the modularity of the sensor surface permits facile adaptation for disease-specific diagnostics by substituting the capture ligands (e.g., anti-HER2 or anti-EGFR antibodies), thus broadening its translational applicability across various biomarker classes. Consistent with the Minimal Information for Studies of Extracellular Vesicles (MISEV) 2018 guidelines,^34^ the isolated EVs were comprehensively characterized using four orthogonal analytical techniques: Western blotting for protein markers, NTA for size distribution, fNTA for marker-specific validation, and electron microscopy for morphological confirmation.

Herein, EVs were isolated through a microfluidic device as explained in an earlier report.^35^ In this regard, we initially measured the concentration and diameter of particles (including EVs) through scatter and fluorescence detection modes of the NTA instrument. The stock concentrations ranged from 10^8^ to 10^10^ particles/mL (Figure 4a) in scatter mode and were further diluted with PBS to obtain concentrations ranging from 10^4^ to 10^6^ particles/μL (Figure 4b). The mean size of the particles was measured as 262.2 ± 16.8 nm. After fluorescent labeling with Alexa 488-anti-CD63 antibodies, the mean size of the same sample was 85.8 ± 34.7 nm with minor peaks more than 100 nm considered negligible, while the mode was 32.9 ± 1.1 nm, consistent with the main sample population in the NTA (Figure 4a,b). This apparent decrease in size following antibody labeling is consistent with observations reported in previous studies.^36–38^ The reduction in measured size is not due to physical shrinkage but would be potentially attributed to the nature of fNTA detection. Briefly, fluorescent antibodies selectively bind to tetraspanin markers (e.g., CD63) that are more abundant on smaller EVs like exosomes, resulting in the tracking of a smaller, marker-positive subpopulation, while larger, marker-negative vesicles remain undetected in fluorescence mode. Additionally, the use of a fluorescence filter excludes larger scatter-dominant particles, which otherwise overshadow smaller vesicles in the scatter mode. Similar trends have been reported by Długolecka et al., who observed that tetraspanin-positive EVs from plasma and BALF showed mean diameters of ∼100–116 nm compared to ∼172 nm in total scatter mode.^36^ In parallel, Thane et al. observed a smaller size when using quantum dot-labeled CD9 antibodies.^37^ Dragovic et al. also reported that NDOG2-positive placental EVs peaked around 100 nm in fluorescence mode versus a broader, larger distribution in scatter mode.^38^

**Figure 4:**
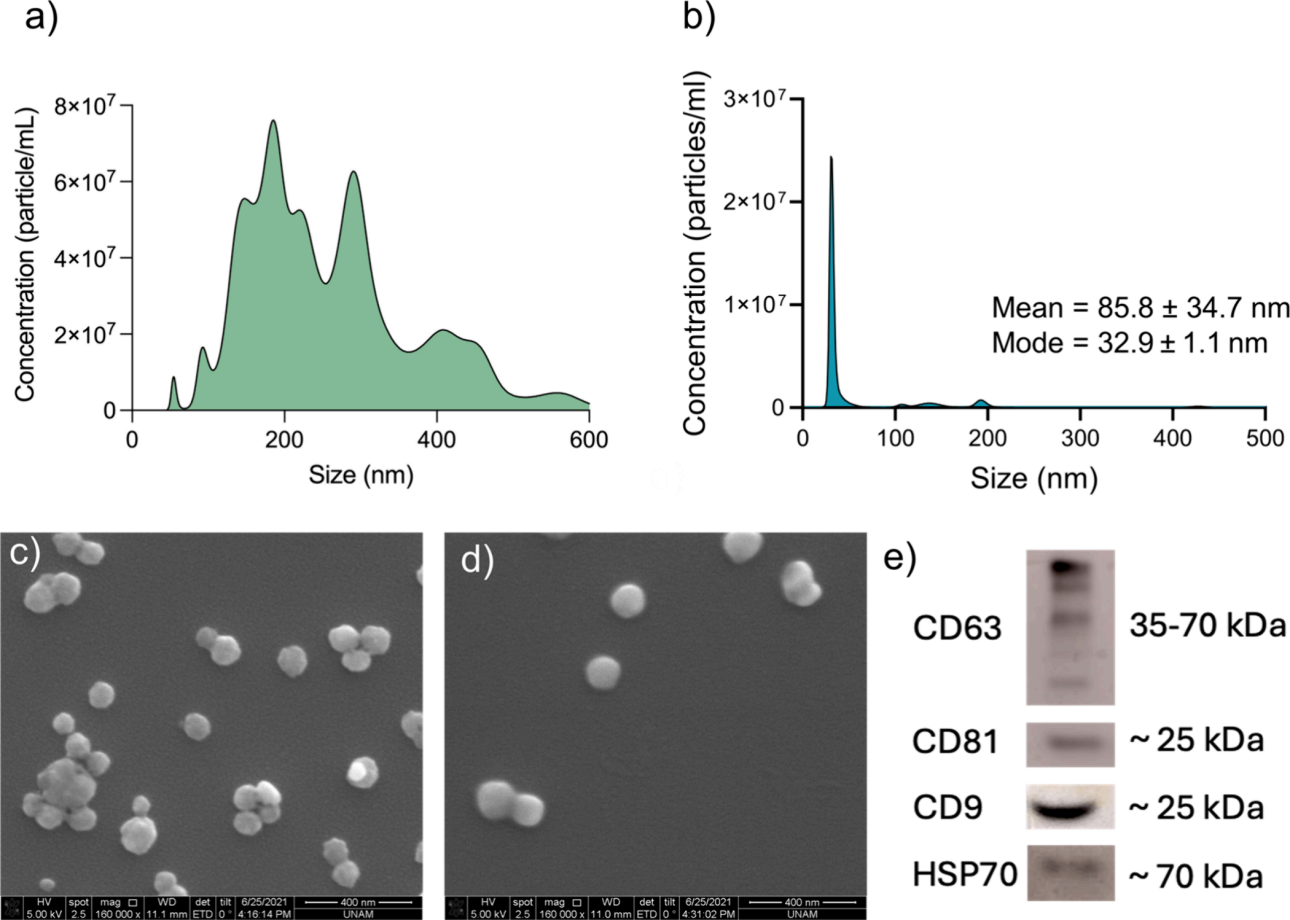
Characterization of EVs. (a) The NTA results of isolated EVs show a concentration of 1.42 × 10^10^ particles/mL with a mean diameter of 262.2 ± 16.8 nm. (b) The f-NTA was performed with Alexa Fluor 488-conjugated anti-CD63 antibodies to confirm the presence of CD63-positive EVs, with a detected concentration of 1.96 × 10^8^ EVs/mL, a mean size of 85.8 ± 34.7 nm, and a mode size of 32.9 ± 1.1 nm. (c–d) Representative SEM images of isolated EVs highlighting their morphology. (e) Western blot analysis of EVs confirming the presence of tetraspanin markers (CD63, CD81, and CD9) and the cytosolic marker—HSP70.

In this study, two distinct approaches were employed to quantify particle concentrations: (i) NTA in scatter mode, which detects all particles in the sample, and (ii) NTA in fluorescence mode (fNTA), which selectively detects EVs via surface marker labeling. In scatter mode, NTA quantifies all nanoparticulate entities—including EVs, lipid particles, and other aggregates—yielding a total particle count reported in particles/μL. In contrast, fNTA enables specific detection of EVs by targeting surface proteins such as CD63, with concentrations reported as EVs/μL. As aforementioned, the specific EV concentration was approximately 72.45-fold lower than the total particle count due to the selective nature of fNTA (Figure 4a,b). Moreover, SEM analysis of the isolated EVs revealed predominantly intact and morphologically spherical particles. Notably, a subset of EVs appeared aggregated, likely due to intervesicular lipid–protein interactions and dehydration-induced aggregation artifacts, as commonly observed in SEM-based EV characterization (Figure 4c,d).^35^

In addition to the size and shape characteristics, protein characterization was performed with Western blot analysis. Tetraspanins (i.e., CD63, CD81, and CD9) are located on the membrane of EVs.^34,39^ In this regard, CD63, CD81, and CD9 were investigated as markers for their identification. In the literature, CD63 is mostly observed as a smear locating between 30 kDa and 60 kDa due to high glycosylation.^40^ CD9 appears as a band at approximately 25 kDa,^41^ whereas the corresponding band of CD81 appears at 20–28 kDa.^40^ As per our results, we observed a smear band for CD63 protein within the range of 35–75 kDa (Figure 4e), and CD81 and CD9 were visible at ∼25 kDa (Figure 4e). In addition to surface markers, heat shock protein 70 (HSP70)−cytosolic protein−was employed as an internal marker for EV characterization. HSP70, typically observed at ∼70 kDa in immunoblotting assays, is a well-established indicator of vesicular origin,^41^ corroborating our results as shown in Figure 4e. Overall, we demonstrated the successful isolation of EVs for further experiments.

For EV detection, we employed an optimized protocol involving the controlled formation of NIs and the integration of AuNPs to enhance the plasmonic sensitivity. In the detection assay, anti-CD81 antibodies were immobilized on the sensor surface to selectively capture EVs via the CD81 protein—a ubiquitous surface protein on EVs.^42^ Since the use of the anti-CD81 antibody is a widely employed method in the literature for detecting EVs,^43,44^ we decorated our sensors with this antibody as a proof-of-concept study. While we do not aim to distinguish different EVs, our platform also has the potential to detect various EVs by simply altering the antibodies only in our surface chemistry approach. As a surface chemistry approach, we initially decorated the bare sensors with chemical linkers (MUA, EDC/NHS), protein G, and anti-CD81 antibodies to capture a fixed concentration of EVs (10^5^ particles/μL) (Figure 5a–j). PBS was passed to the sensors as a baseline, and the signals were recorded with our in-house software. Detecting EVs on the bare gold-top sensor caused 0.94 nm ± 0.29 nm of red-shift in the resonance wavelength (Figure 5a), which was higher than the bare version of silver-top sensors (0.77 nm ± 0.34 nm) (Figure 5d).

**Figure 5:**
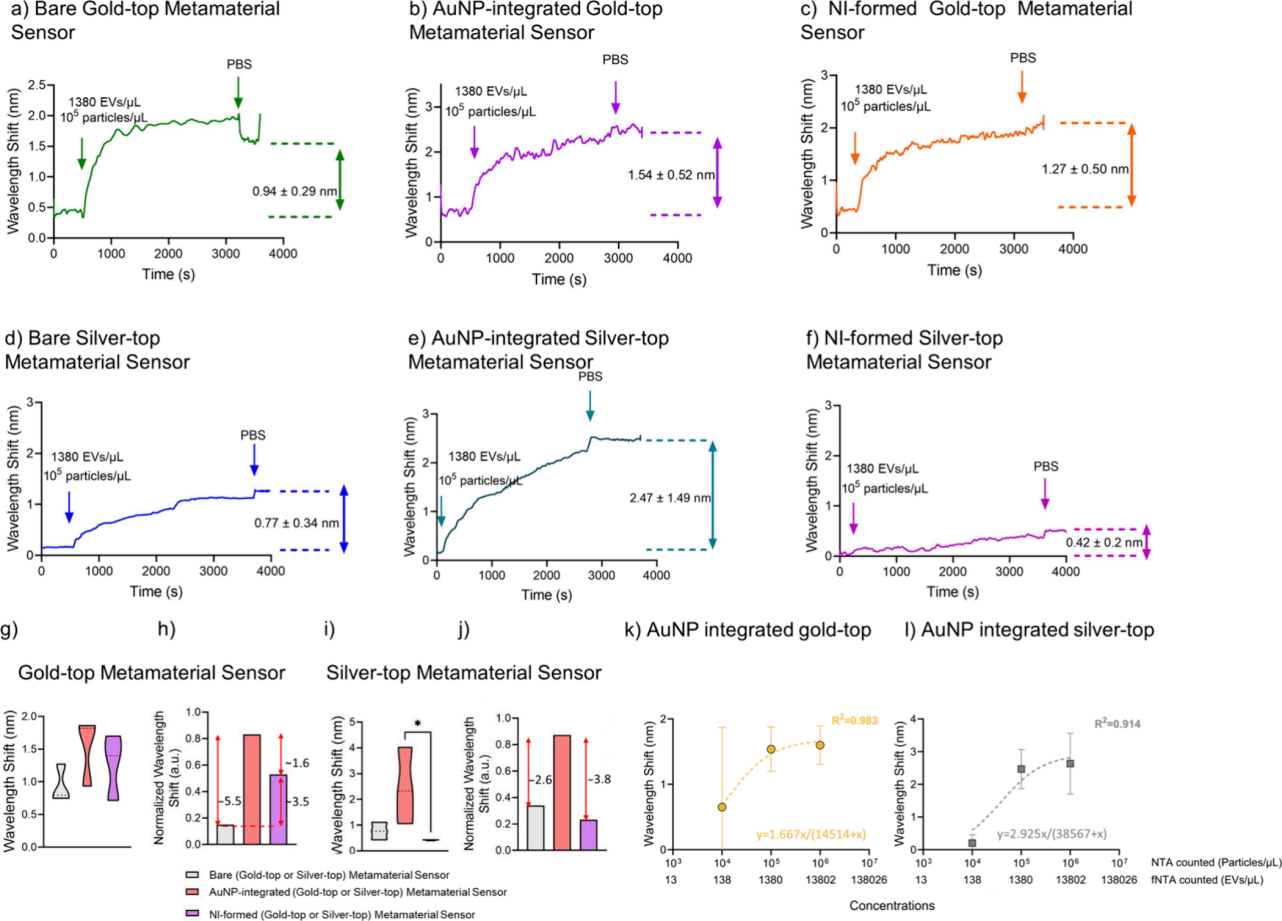
Real-time detection of EVs on metamaterial sensors. Responses are shown for (a–c) bare, AuNP-integrated, and NI-formed gold-top sensors, as well as (d–f) the corresponding silver-top variants. (g) Violin-box plots for gold-top sensors show no statistically significant differences according to a nonparametric Kruskal–Wallis test (*n* = 3, *p* = 0.3607). (h) Normalized data (0–1) illustrate fold changes among gold-top variants. (i) According to a nonparametric Kruskal–Wallis assessment, different versions of silver-top sensors reveal statistical differences, as shown with an asterisk (*n* = 3, *p* = 0.0107). (j) Normalized data (0–1) illustrate fold changes among silver-top sensors. (k, l) Dose–response curves are demonstrated for antibody-modified AuNP-integrated gold- and silver-top sensors exposed to varying EV concentrations (10^6^ particles/μL (equivalent to 13802 EVs/μL) to 10^4^ particles/μL (equivalent to 138 EVs/μL)).

We next considered detecting EVs during signal enhancements achieved by constructing either an NI or AuNP on the metamaterial sensors. Initially, EV detection was assessed with AuNP-integrated gold- and silver-top sensors, which were prefunctionalized with protein G for enabling proper orientation of antibodies. In the case of AuNP integration, the average shifts were calculated as 1.54 nm ± 0.52 and 2.47 nm ± 1.49 nm for the gold-top and the silver-top sensors, respectively (Figure 5b,e). In the case of NI formation, the same conditions were tested and the average redshifts on the resonance were calculated as 1.27 ± 0.50 nm for the gold-top sensors and 0.42 ± 0.2 nm for the silver-top sensors (Figure 5c,f). As elaborated above, NI-formation did not provide high sensitivity for silver-top sensors, and this might be due to the paucity of initial gold structure that would start and/or maximize nanoparticle formation. Moreover, local formation aggregates might impede the sensitivity of the NI-formed silver-top sensors.

To better understand these outputs, we normalized (0–1) the resultant data for each condition and compared the normalized data with that of bare version. For normalization, mean and standard deviation of the data were used to enable consistent comparison across measurements. The NORM.DIST function was applied where appropriate to assess data distribution. All calculations were performed using standard functions on Microsoft Excel software. Considering the gold-top sensors, we observed more than 3.5 (>350%) times of signal enhancements in NI formation but ∼5.5 times (∼550%) of signal enhancements in AuNP-integrated versions. In addition, AuNP-integration to gold-top sensors enhanced the signal ∼1.6 times (∼160%) compared to the NI formation (Figure 5g–h).

Likewise, after normalizing the data (0–1) of silver-top metamaterial sensors and their modified versions, we observed ∼2.6 times (∼260%) of signal enhancements in AuNP integration compared to the bare version, whereas their NI formations resulted in reduction (∼68%) in the signal compared to the bare surface. In addition, AuNP-integration to silver-top sensors enhanced the signal by ∼3.8 times (∼380%) compared with the NI formation (Figure 5i–j). Putting in a nutshell, in situ spatial designs through NI formation and AuNP integration on gold-top sensors provided more signal enhancements compared to their bare versions. Particularly, AuNP-integrated surfaces provided higher wavelength shifts in both silver- and gold-top metamaterial sensors, and no statistically significant differences were observed between these versions (*n* = 3, *p* > 0.05) (Figure S10). Only significant differences were observed between AuNP-integrated silver-top sensors and bare gold-top sensors and between AuNP-integrated silver-top and NI-formed silver-top surfaces (*n* = 3, *p* < 0.05) (Figure S10).

In addition, we expanded our detection range between 10^6^ particles/μL (equivalent to 13802 EVs/μL) and 10^4^ particles/μL (equivalent to 138 EVs/μL). In the case of AuNP-integrated gold-top sensors, we observed sequential changes in the signals from 0.65 to 1.60 nm, whereas AuNP-integrated silver-top sensors resulted in the signals spanning from 0.20 to 2.63 nm while increasing EV concentrations (10^6^ particles/μL (equivalent to 13802 EVs/μL) to 10^4^ particles/μL (equivalent to 138 EVs/μL)) (Figure 5k,l). A broad range in detecting EVs hence leveraged the potential of our system for applications as a practical tool for more accurate sensing. As a proof-of-concept validation, SEM imaging confirmed the successful capture of EVs on the sensor surface, as illustrated in Figure S11.

In these experiments, we systematically analyzed the sensor response to varying EV concentrations, considering both diffusion-limited and affinity-driven interactions. While diffusion constraints dominate at low concentrations, antibody-functionalized surfaces enhance capture efficiency, leading to higher-than-expected surface coverage. Additionally, the nanostructured sensor amplifies local binding through field localization effects, deviating from classical diffusion models. Plasmonic shifts, ideally correlated with bound EV mass, are influenced by spatial distribution and refractive index heterogeneity, which may not scale linearly with concentration. At higher concentrations, deviations from linearity may arise due to (i) surface saturation, (ii) steric hindrance limiting effective binding, and (iii) transient binding-dissociation dynamics affecting the net signal response. Despite these nonidealities, the assay maintains a quantifiable and reproducible dynamic range, as shown in Figure 5k,l. Hence, we evaluated multiple EV concentrations to assess the sensor performance comprehensively.

The limit of detection (LOD) can be determined either empirically or by mathematical modeling. One common approach is based on linear regression, where the instrument response (*y*) is assumed to be linearly correlated with concentration (*x*), following the equation *y* = *a* + *bx*, where *a* is the intercept and *b* is the slope. Based on this model, LOD is calculated using the formula LOD = 3*S*/*b*, where *S* is the standard deviation of the response, estimated from the standard deviation of the *y*-intercept in the regression analysis.^45^ Given that this study serves as a proof-of-concept for EV detection using this platform, experiments were conducted with limited EV concentrations. Therefore, we present the LOD based on actual experimental results, which offers a more realistic evaluation of platform sensitivity. Consequently, the minimum detectable concentration was quantified as 138 EVs/μL, according to the EV quantification using the fNTA.

Compared to conventional sandwich ELISA for HEK293-derived EVs, such as the Vesicure kit, which reports an LOD around ∼1 × 10^7^ EVs/mL (approximately 10^4^ EVs/μL),^46^ and the nanobody-based ELISA by Popovic et al.,^47^ which detects down to 1–2 μg of EV protein per well. Our sensor demonstrates comparable or potentially better sensitivity. Moreover, our platform operates as a label-free system, eliminating the need for secondary antibodies. It features a streamlined workflow with minimal procedural steps (sample loading and washing), thereby avoiding prolonged incubation periods (from 3 h to overnight) and repetitive wash cycles (from 5 to 20 times). When integrated with low-cost, repurposed off-the-shelf substrates and a portable readout device, this configuration underscores the strong potential of our sensor for rapid, simplified, and cost-efficient EV detection—particularly in POC applications.

To further evaluate the sensor’s specificity, surfaces functionalized with antibodies other than anti-CD81 antibody, as well as unmodified control surfaces (without antibody modifications), were tested. The concentration of EVs were set to 10^5^ particles/μL (1380 EVs/μL), and there was a slight increase in the signal; however, when we compared to the anti-CD81 antibody-functionalized surface, this increase was negligible. The EpCAM-modified surface also had a similar increase, which can be explained with potential nonspecific binding (Figure S12,13). This can be easily eliminated with blocking with BSA as we demonstrated earlier.^14^

Regarding comprehensive analytical validation, any interfering factors caused by complex matrices were evaluated while detecting EVs in distinct biological matrices—artificial urine and plasma, in addition to physiological buffers such as PBS. To determine which versions of the sensor to be employed for analytical validation experiments, we took statistical assessments into consideration. Accordingly, while comparing AuNP-integrated silver- and gold-top sensors, there were no statistical differences observed (*n* = 3, *p* > 0.05) (Figure S10). Since there were statistical differences between AuNP-integrated and NI-formed silver-top sensors (*n* = 3, *p* < 0.05) (Figure 5), we continued the experiments with AuNP-integrated versions of sensors in order to detect EVs spiked in complex biological specimens.

The selectivity of our platform was assessed by spiking EVs in artificial urine and plasma samples, containing a plenty of proteins and lipids that would potentially interfere with the sensor surface, and hence, we tested such conditions for evaluating specific binding of EVs (Figure 6). Interestingly, we observed a sharp increase while applying artificial urine to the sensors, which might be caused by the refractive index of ingredients in these specimens. After applying PBS to remove unbound molecules, we observed a sharp decrease, pointing out that this initial fluctuation (increase/decrease) might be caused by the different RI values of artificial urine compared to PBS. Herein, we observed ∼1.30 and ∼1.82 nm for AuNP-integrated gold- and silver-top sensors, respectively (Figure 6a–d). Moreover, we applied artificial urine samples without EVs (control) to both sensors and did not observe any significant shifts compared to the experimental value (10^5^ particles/μL or equivalent to 1380 EVs/μL in artificial urine) (Figure 6b,d).

**Figure 6:**
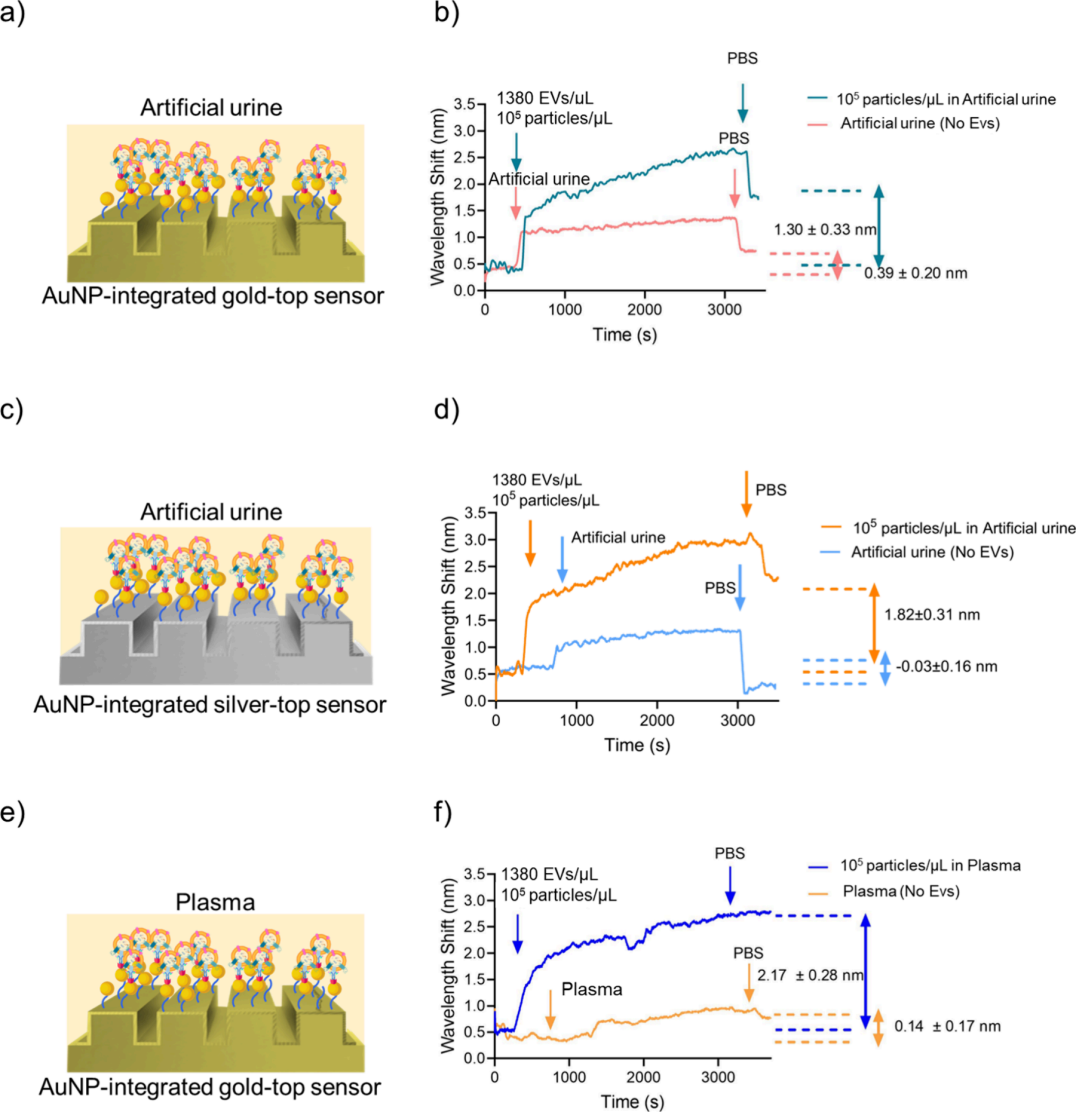
Validation of sensor performance in complex biological matrices. (a–b) AuNP-integrated gold-top sensors detecting 10^5^ particles/μL (equivalent to 1380 EVs/μL) spiked into artificial urine, compared to EV-free control. (c–d) Equivalent evaluation of AuNP-integrated silver-top sensors in artificial urine. (e–f) Analytical performance of AuNP-integrated gold-top sensors detecting 10^5^ particles/μL (equivalent to 1380 EVs/μL) spiked into plasma, with EV-free plasma as control.

To assess the specificity of the sensor platform, we quantitatively evaluated the detection results by calculating the selectivity coefficient (*k*),^48^ as defined in eq 2:

$$k=\mathrm{wavelength}\;{\mathrm{shift}}_{\mathrm{EV}}/\mathrm{wavelength}\;{\mathrm{shift}}_{\mathrm{Artificial}\;\mathrm{Urine}}$$ 2

Accordingly, *k* values were calculated for the AuNP-integrated gold- and silver-top sensor configurations, yielding values of 3.33 and 4.55, respectively. As per the criteria in the literature,^49–51^ a *k* value greater than 1 indicates high binding specificity. These results confirm that EVs were selectively captured on the sensor surfaces, while artificial urine did not elicit significant nonspecific signals.

Beyond artificial urine analysis, we conducted an extended analytical validation by detecting EVs (10^5^ particles/μL; equivalent to 1380 EVs/μL) spiked into plasma using the AuNP-integrated gold-top sensor. Following the binding step, the sensor surface was rinsed with PBS to remove the unbound materials. A distinct plasmonic wavelength shift of approximately 2.17 ± 0.28 nm was observed (Figure 6e–f), pointing out the successful EV capture. As a negative control, plasma samples without EVs were similarly analyzed, resulting in negligible spectral shifts compared to those of the EV-containing samples (Figure 6f). The selectivity coefficient for this experiment was calculated as 15.5, further substantiating the sensor’s ability to discriminate specific EV signals from complex biological media with minimal nonspecific interference.

Overall, we comprehensively validated the analytical performance of metamaterial sensors with artificial urine and plasma samples as model biological conditions. As per all the results, the detection performance was not significantly hindered with complex matrices (Figure 6), and we obtained comparable results with the ones performed in PBS conditions (Figure 5).

In addition, current surface chemistry offers a versatile platform capable of immobilizing diverse antibodies for various biotargets. However, further considerations remain regarding the long-term stability of surface functionalization, particularly for POC testing (POCT). Notably, the present surface design lacks protective stabilizing layers—such as silk fibroin^52^ or trehalose^53^—which are known to enhance the durability of antibody immobilization under ambient conditions. Future efforts will focus on incorporating such stabilizing coatings to extend the storage life and preserve bioactivity. Given that POCT often involves the direct application of complex biological fluids, such as whole blood or saliva, minimizing sample preparation is essential. These fluids typically necessitate preprocessing steps—including dilution, filtration, or on-chip separation—to reduce fouling and nonspecific binding. In this study, we validated our sensor’s performance by using spiked EVs in artificial urine and plasma to simulate complex matrices. Future work will aim to use complex biological fluids directly without preprocessing steps and enable testing with unprocessed clinical samples (e.g., whole blood or stool). Hence, this approach would allow for a more accurate assessment of the platform’s practical utility in the real-world POC settings.

## 3 Conclusions and Outlook

In this study, we aim to repurpose commercially available optical disks as substrates for metamaterial-based plasmonic sensors, thereby reducing the fabrication cost, complexity, and processing time. Through the engineering of spatially controlled nanoarchitectures—including in situ-formed NIs and ex situ immobilized AuNPs—we generated tunable plasmonic hotspots that markedly enhanced the optical response of the sensor. A PS base layer was derived from optical disk material via a combination of physical processing and chemical etching, yielding nanograting structures suitable for plasmonic applications. A plasmonic alloy layer (Ti, Ag, and Au) was subsequently deposited through thermal evaporation under the controlled chamber conditions. This fabrication strategy dramatically reduced sensor production costs: $27 for a silver-top disk (30 sensors, $0.90 per sensor) and $45 for a gold-top disk ($1.50 per sensor). In comparison to conventional nanograting fabrication using e-beam lithography (∼$7000 per 4 in. wafer), this approach offers a 260-fold cost reduction for silver-top and a 154-fold reduction for gold-top configurations. Additionally, the overall processing time was reduced from ∼80 h (e-beam lithography) to only 5 min for the physical and chemical preparation steps, representing a 960-fold improvement. Metal deposition and spatial patterning steps were excluded from this time-cost analysis as they are common to both methods.

Second, beyond simplified and cost-effective fabrication, our metamaterial sensors maintain a persistent nanograting structure that facilitates efficient light propagation. This design obviates the need for complex optical pathways involving mirrors, prisms, or angular configurations, enabling the development of compact and portable sensor systems that are well-suited for POC applications.

Third, from an analytical perspective, the primary objective was to enhance sensor sensitivity by amplifying localized E-fields at plasmonic hotspots formed within nanogaps between metallic features. This was achieved by spatially integrating AuNPs and NIs via facile chemical processes including solution-based incubation onto biomolecular (dielectric) layers. Using these methods, we fabricated six distinct metamaterial sensor variants: bare gold-top, NI-formed gold-top, AuNP-integrated gold-top, bare silver-top, NI-formed silver-top, and AuNP-integrated silver-top. These exhibited refractive index sensitivities ranging from 405.47 RIU/nm (bare gold-top) to 546.42 RIU/nm (NI-formed gold-top), with spatially engineered designs providing up to a 5.5-fold sensitivity enhancement relative to their bare counterparts.

In addition, we have validated the analytical performance of these sensors by detecting EVs spiked in a range of biologically relevant media including physiological buffer, artificial urine, and plasma. To the best of our knowledge, this is the first demonstration of in situ spatial configuration control on DVD-derived sensors for EV detection across multiple sample types. From a fabrication standpoint, our approach offers a facile and innovative production scheme encompassing: (i) direct utilization of polycarbonate DVD substrates without replication or molding, (ii) in situ formation or ex situ integration of plasmonic nanostructures (either NIs or AuNPs), (iii) minimal and low-temperature processing steps, and (iv) a label-free, nonfluorescent detection strategy. Our system also enables real-time monitoring through a user-friendly interface, eliminating the need for complex data postprocessing.

To quantify the EV concentrations, both NTA and fNTA were employed. Based on raw NTA data, the platform demonstrated a detection limit of 10^4^ particles/μL, equivalent to ∼330 fg/μL, which was calculated by recent reports. This conversion relies on Rosa-Fernandes et al.'s study since they presented that 3 × 10^10^ particles would correspond to 1 μg of proteins for high vesicular purity and 2 × 10^9^ to 2 × 10^10^ particles/μg would correspond 1 μg of proteins for low purity.^54^ In an earlier study, the detection limit was 8.7–16 μg/mL of lipid vesicles.^55^ In addition to aforementioned reports, Küçük et al. presented a comprehensive review about optical biosensing for EV detection.^56^ The KeyPLEX platform, for instance, introduces electrokinetically enhanced nanoplasmonic sensing, which effectively overcomes diffusion-limited target capture and enables the detection of rare EVs directly from plasma.^57^ Notably, it achieves an LOD of ∼30 EVs (∼1 EV/μL) when it utilizes an external potential (active mode). Nevertheless, its reliance on an external electric potential, while central to its ultrasensitive performance, poses challenges for portability and complicates seamless integration into POC settings. Similarly, a dual gold nanoparticle–amplified SPR aptasensor has been reported for cancer exosome detection, achieving an impressive LOD of as low as 5 × 10^3^ exosomes/mL (5 exosomes/μL).^58^ However, the necessity for dual nanoparticle labeling and intricate surface functionalization introduces additional complexity and cost, thereby limiting their practical translation into streamlined POC assays. In another noteworthy contribution, Li developed a WS_2_-supported gold nanobipyramid-modified optical microfiber sensor capable of detecting prostate cancer exosomes with exceptional sensitivity, achieving an LOD of 23.5 particles/mL (less than 1 EVs/μL) in PBS and 570.6 particles/mL (less than 1 EVs/μL) in serum.^59^ While this performance surpasses that of many state-of-the-art platforms, the requirement for multistep nanofabrication may hinder scalability and impede its routine implementation in clinical diagnostics. Moreover, fNTA measurements revealed an ∼72.45-fold lower effective EV concentration, yielding an actual detection limit of approximately 138 EVs/μL (Table S5). Therefore, we report three distinct LOD values corresponding to raw particle counts reliant on NTA results, literature-based mass calculations, and actual EV quantification through fNTA measurements: 10^4^ particles/μL, approximately 330 fg/μL, and 138 EVs/μL, respectively. Furthermore, in Table S5, our primary emphasis was placed on optical-based sensing mechanisms due to their label-free detection capability, ease-of-miniaturization, and compatibility with real-time monitoring. Nevertheless, alternative platforms such as magneto-electrochemical sensors have also been explored for EV detection. For instance, Park et al. reported a magneto-electrochemical system that enables the isolation of tumor-derived EVs directly from patient plasma by employing immunomagnetic beads conjugated with target-specific antibodies. Following magnetic enrichment, the captured EVs were electrochemically profiled on a nanostructured electrode surface. Although this approach achieves impressive detection sensitivities—down to approximately 10^4^ EVs/mL—it necessitates a series of complex steps, including sophisticated nanofabrication and immunomagnetic separation procedures and the simultaneous use of three different tetraspanins. These addtional requirements may increase the operational complexity, cost, and processing time, potentially limiting the scalability and practical applicability of such platforms in routine clinical diagnostics.^60^

In summary, our work presents an innovative, cost-efficient, and scalable strategy for fabricating plasmonic metamaterial sensors using repurposed optical disk substrates. The proposed platform addresses key challenges associated with the ASSURED (Affordable, Sensitive, Specific, User-friendly, Rapid and Robust, Equipment-free, and Deliverable) criteria, including reductions in fabrication cost and complexity, enhanced analytical performance, miniaturized hardware requirements, and compatibility with POC diagnostic workflows. The detection of EVs in this study serves as a representative application to assess the feasibility, specificity, and analytical performance of this platform. Accordingly, this work represents a critical advancement toward the development of a novel biosensing approach suitable for deployment in POC settings, with the long-term goal of enabling integration into clinical diagnostics and research workflows. While the current findings provide compelling evidence of the sensor’s functionality and potential, further validation using clinically relevant samples is essential to establish its translational utility and clinical applicability. Moreover, the modularity and adaptability of the platform suggest that its applicability would be extended to a variety of complex biological matrices, including whole blood, bronchoalveolar lavage fluid, saliva, and sweat. Hence, a separate study focused on clinical validation would involve assessing these sensors using patient-derived samples, thereby directly addressing a specific clinical question.

## 4 Experimental Section

### 4.1 Materials

Methanol (99.7%), poly-l-lysine (PLL), 3-mercapto­propanyl-*n*-hydroxy-succinamide ester (3-MNHS), sodium citrate dihydrate, gold­(III) chloride trihydrate (HAuCl_4_ · 3H_2_O), and hydroxylamine hydrochloride were purchased from Sigma-Aldrich. Absolute ethanol (99.9%) and glycerol (85%) were obtained from Isolab Chemicals. The other organic solvents, including ethanol (96%), isopropanol alcohol (99.5%), and acetone (99.5%), were obtained from Soltek, Lobal Chemie, and Birpa, respectively. Phosphate saline (PBS) tablets were purchased from Biomatik. HP brand DVDs were used as a PS template. In developing metamaterial sensors, gold (999.9) and silver (999.0) were obtained from Istanbul Gold Refinery. All chemicals were used without further purification, and deionized water was used throughout. In the cell culture studies, penicillin/streptomycin, l-glutamine, DMEM/F-12 (Dulbecco’s Modified Eagle Medium/Nutrient Mixture F-12, Biowest), and Trypsin-EDTA were purchased from Gibco. Fetal bovine serum (FBS) was purchased from Biowest.

### 4.2 Sensor Fabrication

Optical disk surfaces have intrinsic nanoperiodic structures in a grating format, and therefore, they were utilized as template PS for the fabrication.^61^ First, commercial optical disks consist of multilayer structures, including polycarbonate plastic surface (top layer), adhesive layer, metal layer, photoresist layer, and, at the bottom, polycarbonate plastic layer. Accordingly, they were removed properly.^14^ The remaining dyes are removed with a mixture of ethanol and methanol (1:1; v:v). Then, the PS was chemically etched with a mixture of acetone and isopropanol (1:4; v:v) for 60 s and then washed with distilled water and dried under compressed air. The periodicity of the nanograting structure, defined by the grating width and spacing, could be tuned by adjusting the etching time. According to our earlier study, we observed that a 60 s etching process provided higher responses to the bulk changes and binding events.^14^ Specifically, longer etching durations tend to increase the spacing between the grating features, which directly influences the optical resonance and signal response by modifying the effective refractive index around the grating. This demonstrates that careful optimization of the etching parameters is essential to maintaining the desired nanograting morphology and ensuring consistent sensor performance. After etching, PS was coated with 10 nm of titanium, 30 nm of silver, and 15 nm of gold sequentially by executing a thermal evaporation under 2 × 10^–6^ Torr of pressure to fabricate a gold-top sensor. For the silver-top sensors, the PS was coated with 65 nm of silver only with thermal evaporation (Midas, Vaksis, Turkey) under 6 × 10^–6^ Torr of pressure. Once the sensors were fabricated, they were cut into 1.5 cm × 1.5 cm by using scissors.

### 4.3 Gold Nanoparticle Synthesis

AuNPs were synthesized through a seeding reaction as explained in the literature.^28^ Briefly, 60 mg of sodium citrate dihydrate was dissolved in 90 mL of distilled water and boiled for 10 min under vigorous stirring. Afterward, 6 mg of HAuCl_4_·3H_2_O was introduced into the solution and kept boiling for 30 min. This solution was cooled down to 90 °C, and then 25 mM sodium citrate (3 mL) and 1 mM HAuCl_4_·3H_2_O solution (3 mL) were added to the seed solution, followed by boiling for 30 min. Finally, the solution was allowed to cool to room temperature and stored at +4 °C until further use.

### 4.4 Fabrication of Gold Nanoparticle-Integrated Sensors

The gold-top and silver-top sensors were cleaned with ethanol under sonication for 5 min. Later, they were dipped into 0.5 mg/mL PLL solution and incubated overnight at +4 °C for the formation of a biomaterial adlayer. After the incubation, the surfaces were washed with distilled water and dried in compressed air. The surfaces were then placed in 1:10 diluted gold nanoparticle solution (0.200 OD (au) at 526 nm of resonance) and incubated overnight at +4 °C. The surfaces were then washed with distilled water and dried again to integrate with a microfluidic chip.

### 4.5 NI Formation on the Sensors

As detailed above, PLL as a biomaterial adlayer was initially developed on metamaterial sensors. For the formation of NI on the surfaces, 1 mM HAuCl_4_ (in water) and 1 mM HONH_2_·HCl (in water) solutions were prepared as stock solution. Before applying the seeding solution to the PLL-modified sensors, each solution was diluted to 20 μM. Afterward, equal volumes of HAuCl_4_ (20 μM) and HONH_2_·HCl (20 μM) were mixed. These surfaces were placed into the seeding solution and incubated for 5 min followed by a washing step with distilled water and allowed to dry for integrating with a microfluidic chip.

### 4.6 Fabricating Microfluidic Chips

The microfluidic chips were composed of (i) poly­(methyl methacrylate) (PMMA, 2 mm thickness), (ii) double-sided adhesive film (DSA, 50 μm thickness), and (iii) a metamaterial sensor (Figure S6e,f). The PMMA layer had the inlet and outlet ports (0.67 mm in diameter), whereas DSA layer was employed as an adhering layer to connect the PMMA layer and sensor. In addition, the DSA layer contains channels for sample flow over the sensor. The channel length, width, and height are 9 mm, 3 mm, and 50 μM, respectively. The chips were designed using RDWorks software, and accordingly, both PMMA and DSA layers were cut with a laser cutter system (LazerFix, Turkey).^62–64^ The microfluidic modules were assembled with the sensor surfaces by peeling the second layer of the DSA film. Samples were introduced through tubings (0.32 mm of inner diameter, 0.76 mm of outer diameter, and polytetrafluoroethylene (PTFE), Adtech, United Kingdom), where they were epoxied to the inlet and outlet ports. After assembly, the microfluidic module was connected to a syringe pump to achieve sample flow through the channels. The flow rate (10 μL/min) was operated with a syringe pump system (New Era Pump Systems, Inc., NY., United States).

### 4.7 Measuring Bulk Sensitivity

Various glycerol solutions spanning from 0% (only distilled water) to 70% (prepared in distilled water) were employed to benchmark the analytical performance of the sensors. Monitoring real-time data was carried out using our in-house MATLAB GUI-based interface, whereas the end-measurements were employed on Thorlabs OSA software. The baseline was recorded with distilled water, and each glycerol concentration was sequentially applied to the sensors, starting from the lowest to the highest concentrations.

### 4.8 Surface Functionalization

In this study, EVs were captured and detected via antibody-functionalized sensor surfaces. Prior to functionalization, the bare gold- and silver-top sensors were thoroughly cleaned and subsequently modified with a thiol-terminated self-assembled monolayer (SAM) using 10 mM 11-mercaptoundecanoic acid (MUA) in ethanol. The bare surfaces were incubated overnight under dark conditions. The sensors were later rinsed with ethanol and dried in compressed air. PMMA microfluidic chips were subsequently placed on top of the bare sensors with a DSA layer. Later, 100 mM:50 mM of EDC: NHS mixture prepared in 50 mM MES buffer (pH 6.0) was introduced to microchannels through tubing and incubated for 1 h at room temperature. These activated esters (EDC/NHS) readily react with primary amine groups on protein G, enabling its covalent immobilization on the sensor surface. The immobilized protein G serves as an orienting scaffold for the Fc-specific attachment of IgG-class capture antibodies, ensuring optimal antigen-binding site exposure and enhancing the subsequent EV capture efficiency. The microchannels were washed with PBS to remove any unbound moieties, and then 100 μg/mL of protein G (in PBS) was incubated overnight at +4 °C to ensure proper orientation of antibodies on the sensors. In this experiment, anti-CD81 antibodies (50 μg/mL prepared in PBS) were decorated on the sensors by incubating for 3 h at +4 °C. After the incubation, we simply washed the sensors with PBS (300 μL) to remove any unbound moieties and incubated with the EV sample. In contrast to the bare sensor surfaces, the NI-formed and AuNP-integrated sensors were first functionalized with PLL to facilitate the nanoparticle or nanoisland formation. After the integration of AuNPs or formation of NIs, additional surface modification steps were carried out to enable specific EV capture. EVs were selectively captured through the interaction between CD81 markers on their membranes and the immobilized antibodies on the sensors. To achieve this, protein G (100 μg/mL in PBS) was introduced into the microchannels and incubated overnight at +4 °C to ensure stable attachment. Next, anti-CD81 antibodies (50 μg/mL in PBS) were applied and incubated for an additional 3 h at +4 °C to bind via protein G. After incubation, the sensors were gently rinsed with 300 μL of PBS to remove any unbound proteins or antibodies, ensuring a clean and specific functionalization layer.

### 4.9 Imaging Studies

The bare and NI-formed sensors and EV-captured sensors were analyzed with a scanning electron microscope (SEM, FEI Quanta 200 FEG). Since the surfaces contained a conductive layer, we used the bare and NI-formed metamaterial sensors as is. For imaging the isolated EVs, we initially mixed EV samples with 2% paraformaldehyde (PFA). We then applied this mixture onto a silicon wafer and left it for drying at room temperature. The samples on the wafer were washed with ethanol three times to remove any moieties and dehydrate, and later on, we coated the samples with 5 nm of Au/Pd before the imaging. Besides, atomic force microscopy (AFM, Asylum Research MFP-3D, Oxford Instrument, United Kingdom) was employed for analyzing the surface topography in a quantitative manner. In this regard, AC mode AFM was utilized in air for imaging. Silicon cantilevers (PPP-NCHR, Nanosensors, Switzerland) have a force constant of 42 N/m. The scanning rate was set to 0.6 Hz, and the images were taken from the 2 μm × 2 μm area, which were further analyzed on Gwyddion software without any data manipulations (such as filtering and smoothing). For characterizing AuNPs, TEM analysis (FEI, Tecnai G2 F30, 300 kV) with energy-dispersive X-ray (EDX) spectroscopy was performed. AuNPs were simply dropped on carbon TEM grids (2 μL), and allowed to dry at room temperature before the imaging.

### 4.10 Isolating and Quantifying EVs

EVs were isolated using a double filter-integrated microfluidic chip (200 and 50 nm of pore size) as detailed in the literature.^35^ Briefly, HEK293 cells were cultivated in FBS-free DMEM/F-12 supplemented with 1% penicillin/streptomycin and 1% l-glutamine. At the end of the fourth day of the incubation, the medium (supernatant of culture) was collected. The supernatant was then centrifuged 15 min at 20.000g to remove cell debris and large particles. After the centrifuge, the supernatant was introduced into the microfluidic chip for isolating EVs. After isolation, the size, size distribution, and concentration of EVs were determined using Nanoparticle Tracking Analysis (NTA) (NS300, Malvern Instruments Ltd., Malvern, United Kingdom).

EVs were identified among the analyzed particles using the anti-CD63 monoclonal antibody (MEM-259) conjugated with Alexa Fluor 488 during fluorescence NTA (fNTA) analysis. This method offers additional insights into whether the isolated particles express CD63 (a tetraspanin marker) compared with electron microscopy examinations. To achieve this, 2 μL of anti-CD63 antibody labeled with Alexa 488 (0.26 mg/mL) was mixed with 1 mL of isolated EVs and incubated in the dark at room temperature for 30 min. After incubation, the samples were analyzed by using the NanoSight300 device for the fNTA, with the 488 nm filter activated to determine the size and concentration of CD63-labeled EVs among all particles.

### 4.11 Protein Characterization of EVs with Western Blot Analysis

Once EVs are isolated from the microfluidic chip, we then validated them through Western blot analysis by focusing their surface markers (CD63, CD81, and CD9) and cytosolic marker (HSP70). Here, the isolated EVs were used as intact EVs without any lysis or centrifugation processes for surface proteins. To reach HSP70 proteins, EVs were lysed with RIPA buffer that contained protease inhibitor. This step was followed by centrifugation at 14,000*g* for 15 min (+4 °C), and the supernatant was utilized for the analysis.

### 4.12 Measuring and Collecting Signals on the Sensors

Our sensing scheme relies on tracking changes at the resonance wavelength of the sensor upon binding of molecules. In this regard, we collected reflected light from the sensor during all binding events. A portable setup (Figure S6) was designed with three main components, including (i) optical components (cosine corrector, lenses, linear polarizer, beam splitter, illuminator input, and iris), (ii) portable CCD spectrometer (Thorlabs, CCS175/M), and (iii) a light source in the visible range (400–1300 nm operating range).

The optical pathway of the measurement starts with delivering light from the source to the sensor through optical parts (lens, linear polarizer (510–800 nm), optical aperture, and beam splitter (50:50) in order). Once light passes the aperture, 50% of the light reaches the sensor; reflects from the sensor and reaches the spectrometer (500–1000 nm, fwhm <0.6 nm) after the cosine corrector-integrated fiber cable. In the analysis, a background was initially measured by placing a gold-plated mirror (background) or a blank channel (filled with air). When the sample was applied to the microchannels, another spectral measurement (sensor-sample-PMMA layer) was recorded (sample data). The sample data was normalized by dividing the mirror data or subtracted with the signal that was obtained from the sample-applied channel.

For data acquisition and real-time spectral analysis, a custom-designed graphical user interface (GUI) was developed by using MATLAB. The interface was engineered to provide a user-friendly environment that minimizes the need for technical expertise or extensive training. Spectral data were acquired directly from the coupled spectrophotometer and subjected to fifth-degree polynomial fitting to reduce noise artifacts while preserving peak fidelity. Resonance peaks were tracked dynamically within a user-adjustable wavelength window, typically ranging from 550 to 650 nm. The smoothed spectra were analyzed using a built-in peak detection algorithm based on MATLAB’s signal processing tools, enabling real-time identification and visualization of spectral features. All acquired and processed data could be automatically saved and exported in a standardized CSV format for downstream analysis. The automation of all key processes—including acquisition, smoothing, peak extraction, and data export—ensures accessibility and consistency across users, irrespective of prior technical training.

### 4.13 Electromagnetic Simulations of the Metamaterial Sensors

The electromagnetic response of the gold-top metamaterial sensor was modeled using FDTD numerical software (Ansys Lumerical) in 2D geometry.^65^ The simulation substrate was based on two-dimensional (2D) grating structure (width = 400 nm, height = 30 nm, and periods = 740 nm). The gold-top metamaterial sensor design was composed in the following order: titanium (10 nm), silver (30 nm), and gold (15 nm). Furthermore, the silver-top metamaterial sensor was composed of a silver film (65 nm). The optical properties of metal layers were studied using respective Palik models,^66^ and the base polycarbonate layer was set to a constant refractive index value of 1.58. The boundary conditions were adjusted antisymmetric in the *x* direction and perfectly matched layer in the *y* direction. A plane wave with p-polarization was utilized as a source. The refractive index of the simulation medium was chosen as 1.33 for modeling water as the medium. The AuNP-integrated gold-top metamaterial sensor was designed with a single AuNP (diameter of 24 nm, Figure 2c) at the middle of the nanograting structure on 2D geometry, representing a cylindrical structure on 3D geometry. Additionally, the NI-formed gold-top metamaterial sensor design was composed of 2D hemiellipse design on top of the nanograting structure (located at the corners of the nanograting structure, Figure 2e). The dimensions of the 2D hemiellipse design (R1= 85 nm, R2= 24 nm) were optimized using AFM measurements (Figure 2d). Similarly, the AuNP-integrated silver-top metamaterial sensor was designed using different AuNP configurations (diameter of 36 nm) on the silver-top metamaterial sensor (Figure 2g) and the NI-formed silver-top metamaterial sensor was modeled using same dimensions (R1 = 85 nm, R2 = 24 nm) of hemiellipse design on the NI-formed gold-top metamaterial sensor (Figure 2k). The absorption spectra of the metamaterial sensors were numerically calculated (absorption = 1 – reflection – transmission) and compared with experimental measurements (Figure S5). During absorption calculations, the transmission spectrum was set to zero value for all wavelengths, since the simulated transmission spectrum was negligible and the experimental measurements were obtained solely from the reflection spectra.

## Supplementary Material



## Notes

Representative topographical height profiles of metamaterial sensor surfaces corresponding to the configurations: Figure S1. Ex situ characteristics of AuNPs and NIs: Figure S2. X-ray photoelectron spectroscopy (XPS) characterization of the chemical composition: Figure S3. Corresponding height profiles of the NI-formed gold-top sensor: Figure S4. Absorption spectra comparing simulation and experimental results for AuNP-integrated and NI-formed metamaterial sensors: Figure S5. Portable measurement system: Figure S6. The impact of NI formation, AuNP integration, seeding concentration, and PLL surface modification on resonance wavelength shifts: Figure S7. Optimization of NI formation and AuNP integration: Figure S8. Comparison of NI formation and AuNP integration on the silver-top sensor: Figure S9. Violin-box plots representing sensor responses for each condition of gold-top and silver-top sensors during the detection of 10^5^ particles/μL: Figure S10. The SEM images of EV capture: Figure S11. Detection of 10^5^ particles/μL (1380 EVs/μL) on the sensor surface in the absence of anti-CD81 antibody: Figure S12. Detection of 10^5^ particles/μL (1380 EVs/μL) on a sensor surface functionalized with anti-EpCAM antibodies: Figure S13. Bulk sensitivity values of the sensors along with corresponding standard deviations: Table S1. Sensor responses for EV detection: Table S2. Sensor responses for EV detection in artificial urine and plasma samples: Table S3. Full width at half-maximum (fwhm) and figure of merit (FOM) values for the sensor configurations: Table S4. Overview of various biosensing platforms reported for EV detection: Table S5 (PDF).
