# Supplementary material for: Spatial Designs on Metamaterial Sensors for Enhancing Signals and Detecting Extracellular Vesicles
Source: ACS Appl Mater Interfaces. 2025 Oct 16;17(47):64048–64. doi: 10.1021/acsami.5c07175 (PMC12673523; doi:10.1021/acsami.5c07175)
Supplement: Supplementary file 1 [file am5c07175_si_001.pdf]

Supporting Information

Spatial Designs on Metamaterial Sensors for Enhancing Signals and Detecting Extracellular Vesicles

Esma Derin[1, 2,], Eylul Gulsen Yilmaz[1,2,], Özgecan Erdem[1],Yusuf Aslan[1,2], Abdullah Kafadenk[1], Süleyman Çelik[3], Ümit Çelik[4], Fatih Inci[1,2,*]

[1] UNAM − National Nanotechnology Research Center, Bilkent University, 06800, Cankaya, Ankara, Turkey.

[2] Institute of Materials Science and Nanotechnology, Bilkent University, 06800, Cankaya, Ankara, Turkey

[3] Sabanci University Nanotechnology and Application Center (SUNUM), Sabanci University, 34956, Orhanli, Tuzla, Istanbul, Turkey

[4] School of Civil Aviation, Firat University, 23119, Merkez, Elazig, Turkey

[*]Corresponding author: finci@bilkent.edu.tr

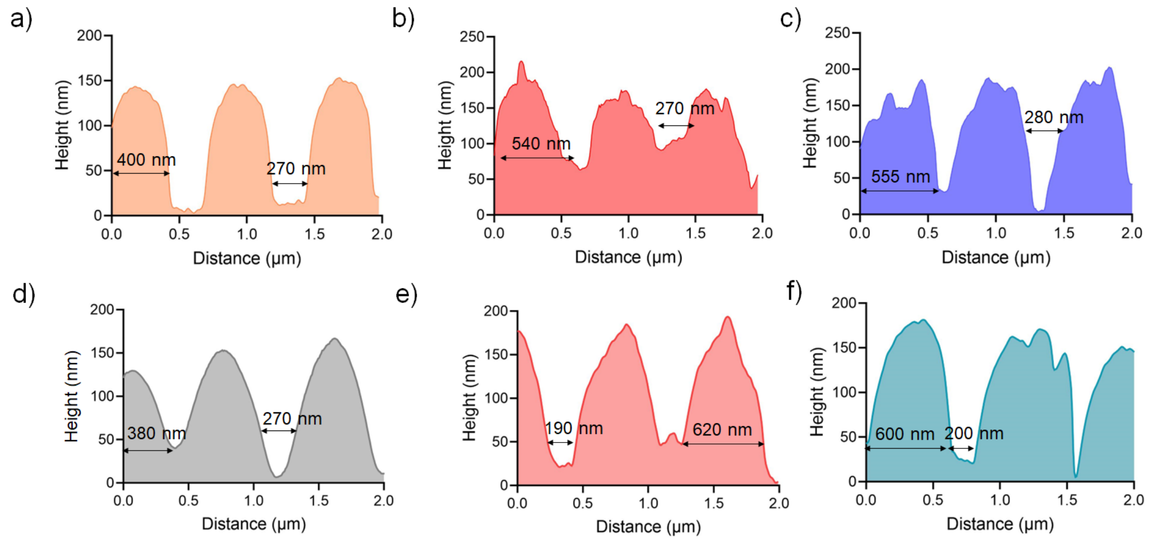

Figure S1. Representative topographical height profiles of metamaterial sensor surfaces corresponding to the configurations illustrated in Figure 1. (a–c) Profiles of gold-top sensors at different fabrication stages: (a) bare substrate, (b) after AuNP integration, and (c) following in situ nanoisland (NI) formation. (d–f) Corresponding profiles for silver-top sensors: (d) bare substrate, (e) after AuNP integration, and (f) following in situ NI formation.

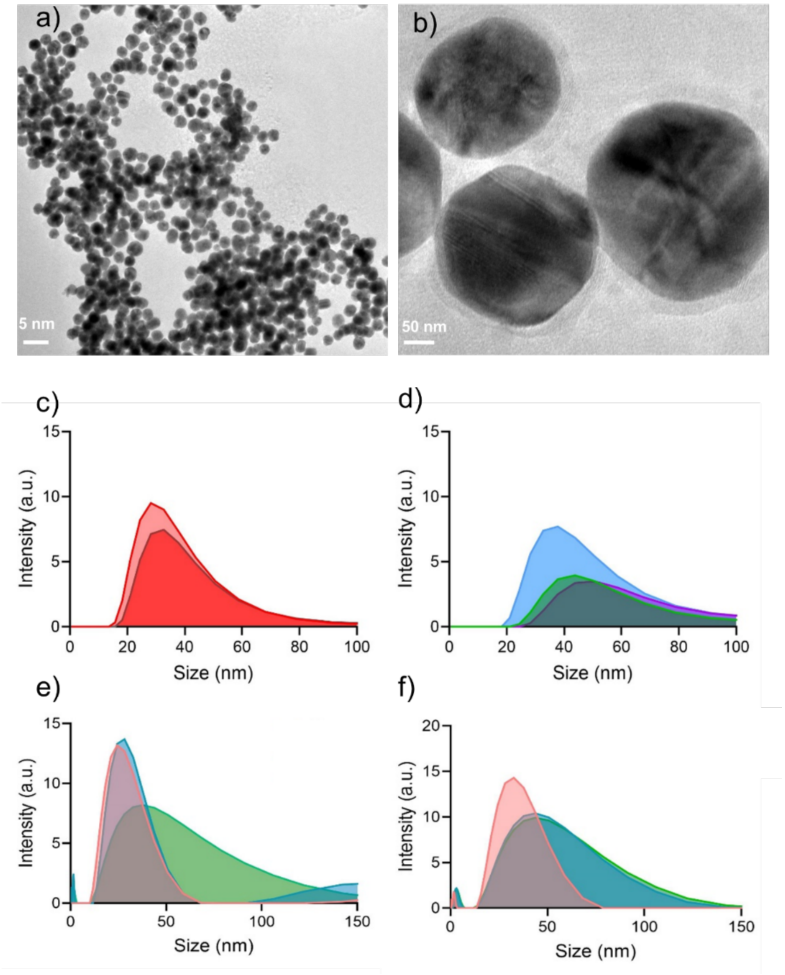

Figure S2. (a–b) Transmission electron microscopy (TEM) images of synthesized gold nanoparticles (AuNPs), confirming their spherical morphology and uniform dispersion. (c) The plot represents the size (diameter) of AuNPs. (d) While applying the seeding solution into AuNP solution, the particle size becomes larger. Zeta measurements are presented for NI-formation in solution while adding (e) 200 μM and (f) 250 μM of seeding solution.

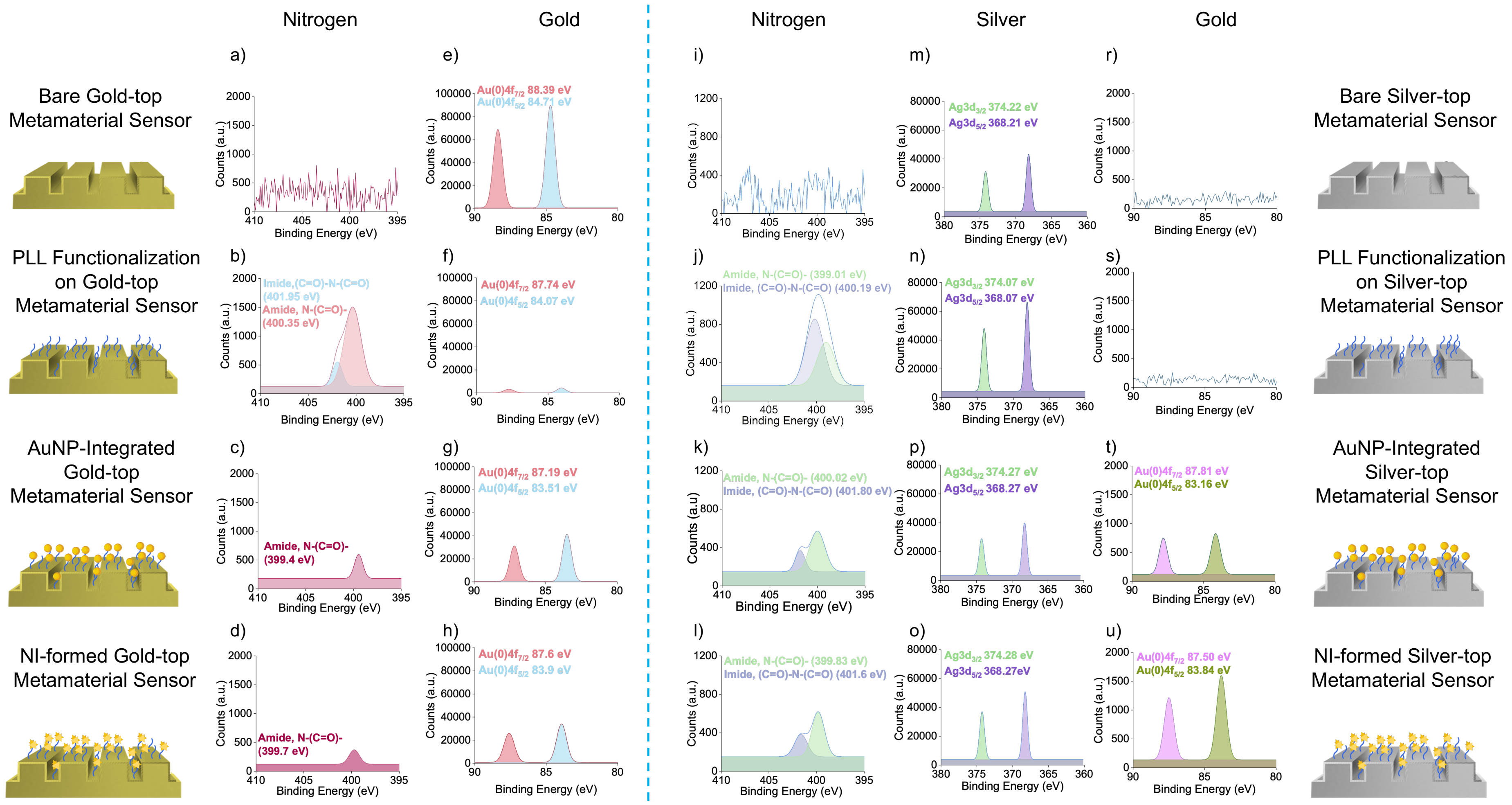

Nitrogen
Gold
Nitrogen
Silver
Gold
Bare Gold-top Metamaterial Sensor
PLL Functionalization on Gold-top Metamaterial Sensor
AuNP-Integrated Gold-top Metamaterial Sensor
NI-formed Gold-top Metamaterial Sensor
Bare Silver-top Metamaterial Sensor
PLL Functionalization on Silver-top Metamaterial Sensor
AuNP-Integrated Silver-top Metamaterial Sensor
NI-formed Silver-top Metamaterial Sensor
a)
b)
Imide,(C=O)-N-(C=O) (401.95 eV)
Amide, N-(C=O)- (400.35 eV)
c)
Amide, N-(C=O)- (399.4 eV)
d)
Amide, N-(C=O)- (399.7 eV)
e)
Au(0)4f7/2 88.39 eV
Au(0)4f5/2 84.71 eV
f)
Au(0)4f7/2 87.74 eV
Au(0)4f5/2 84.07 eV
g)
Au(0)4f7/2 87.19 eV
Au(0)4f5/2 83.51 eV
h)
Au(0)4f7/2 87.6 eV
Au(0)4f5/2 83.9 eV
i)
j)
Amide, N-(C=O)- (399.01 eV)
Imide, (C=O)-N-(C=O) (400.19 eV)
k)
Amide, N-(C=O)- (400.02 eV)
Imide, (C=O)-N-(C=O) (401.80 eV)
l)
Amide, N-(C=O)- (399.83 eV)
Imide, (C=O)-N-(C=O) (401.6 eV)
m)
Ag3d3/2 374.22 eV
Ag3d5/2 368.21 eV
n)
Ag3d3/2 374.07 eV
Ag3d5/2 368.07 eV
p)
Ag3d3/2 374.27 eV
Ag3d5/2 368.27 eV
o)
Ag3d3/2 374.28 eV
Ag3d5/2 368.27eV
r)
s)
t)
Au(0)4f7/2 87.81 eV
Au(0)4f5/2 83.16 eV
u)
Au(0)4f7/2 87.50 eV
Au(0)4f5/2 83.84 eV
Counts (a.u.)
Binding Energy (eV)

Figure S3. X-ray photoelectron spectroscopy (XPS) characterization of the chemical composition of (a–h) gold-top and (i–u) silver-top metamaterial sensors. Briefly, N1s spectra are shown for (a) bare, (b) PLL-modified, (c) AuNP-integrated, and (d) NI-formed gold-top sensors, while Au4f spectra are presented for (e) bare, (f) PLL-modified, (g) AuNP-integrated, and (h) NI-formed gold-top sensors. Likewise, N1s spectra for silver-top sensors are displayed for (i) bare, (j) PLL-modified, (k) AuNP-integrated, and (l) NI-formed samples. Ag3d spectra of silver-top sensors are shown for (m) bare, (n) PLL-modified, (p) AuNP-integrated, and (o) NI-formed conditions. Additionally, Au4f spectra for silver-top sensors are presented for (r) bare, (s) PLL-modified, (t) AuNP-integrated, and (u) NI-formed samples.

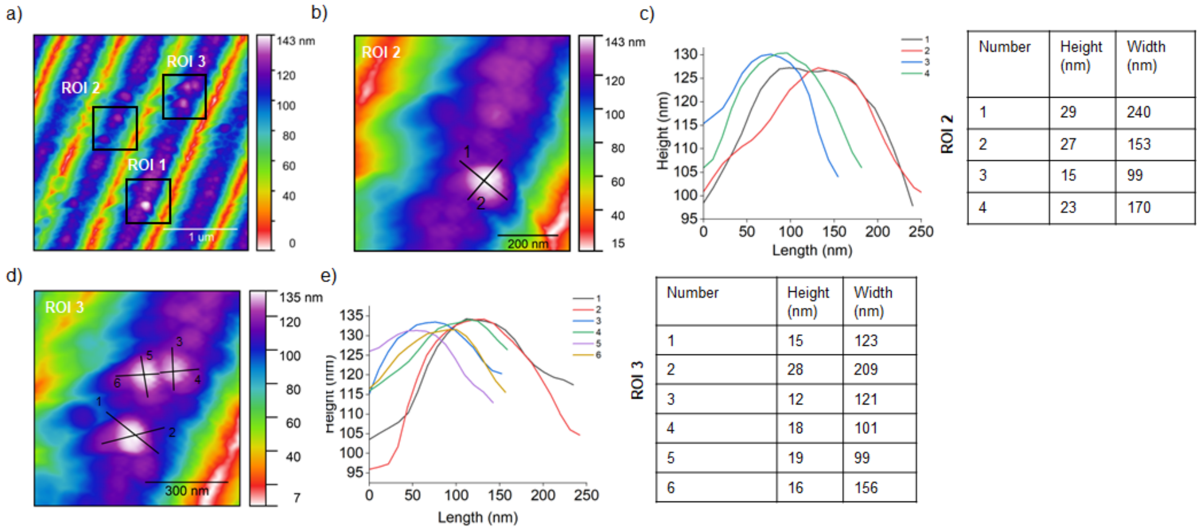

Figure S4. (a) Atomic force microscopy (AFM) image of the NI-formed gold-top sensor. (b) Zoomed-in views of the regions of interest (ROIs) are shown for ROI 2. (c) Corresponding height profiles along the indicated lines in ROI 2 to reveal the topographical features within these areas. (d) Zoomed-in views of the regions of interest (ROIs) are shown for ROI 3. (e) Corresponding height profiles along the indicated lines in ROI 3 to reveal the topographical features within these areas.

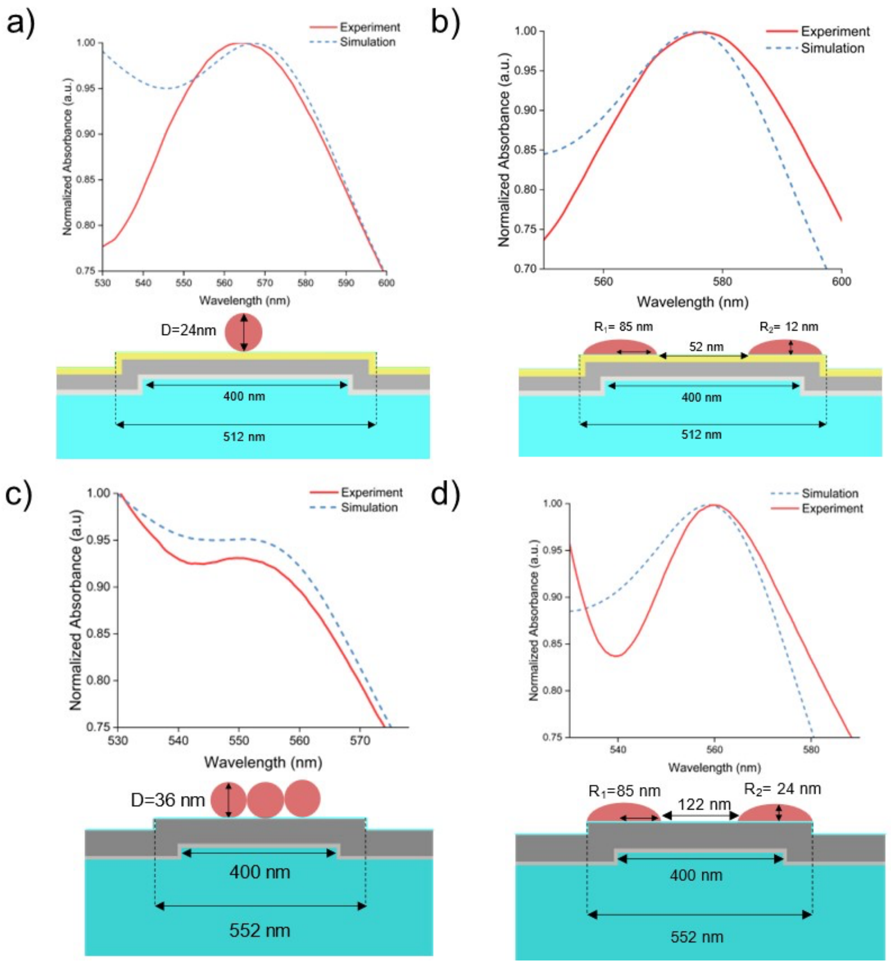

Figure S5. Absorption spectra comparing simulation and experimental results for AuNP-integrated and NI-formed metamaterial sensors. Upper panels show the absorption spectra of (a) AuNP-integrated gold-top, (b) NI-formed gold-top, (c) AuNP-integrated silver-top, and (d) NI-formed silver-top sensors. Corresponding simulation designs of the metamaterial sensors are provided below each reflection spectrum plot.

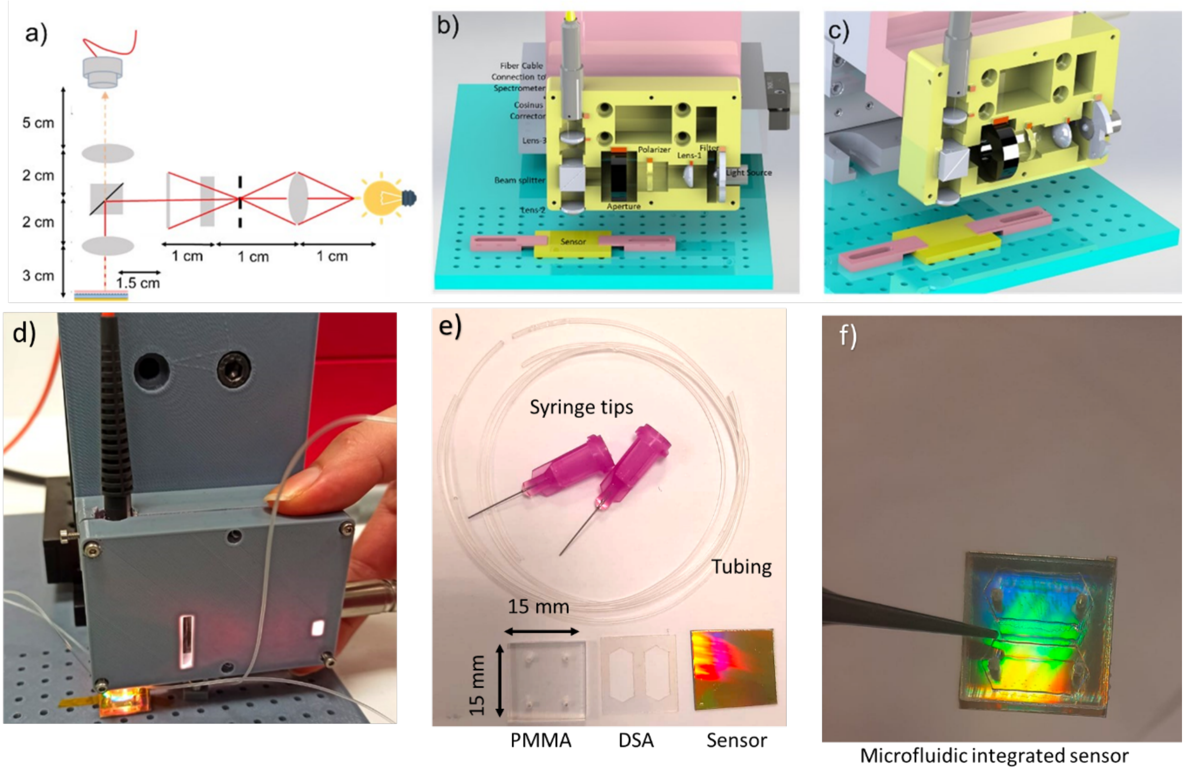

Figure S6. Portable measurement system. (a) Schematic diagram illustrating the optical path within the setup. (b, c) 3D CAD designs of the portable platform. (d) Photograph of the assembled portable platform. (e) Components of the sensor assembly. (f) Microfluidic-integrated sensor surface shown without tubing connections.

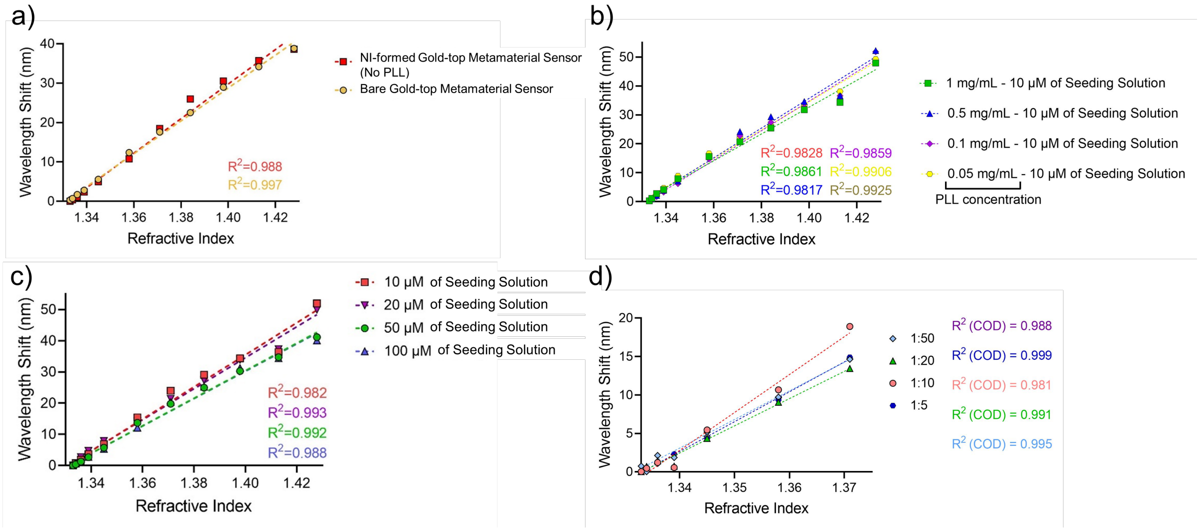

Figure S7. (a) Considering NI-formed gold-top sensors, red-shifts in the plasmonic wavelength are presented for various refractive index in the presence and absence of PLL-modification. (b) The impact of PLL concentrations is evaluated for NI formation, and analytical performance is benchmarked while changing the refractive index of bulk condition through various concentrations of glycerol. (c) The impact of seeding concentration is evaluated while keeping a fixed condition of PLL-modified surface (0.5 mg/mL) for NI formation. The analytical performance is also benchmarked while changing the refractive index of bulk condition through various concentrations of glycerol on these sensors. (d) The effect of different AuNP dilutions is assessed while altering the refractive index of bulk condition through various concentrations of glycerol on AuNP-integrated gold-top sensors.

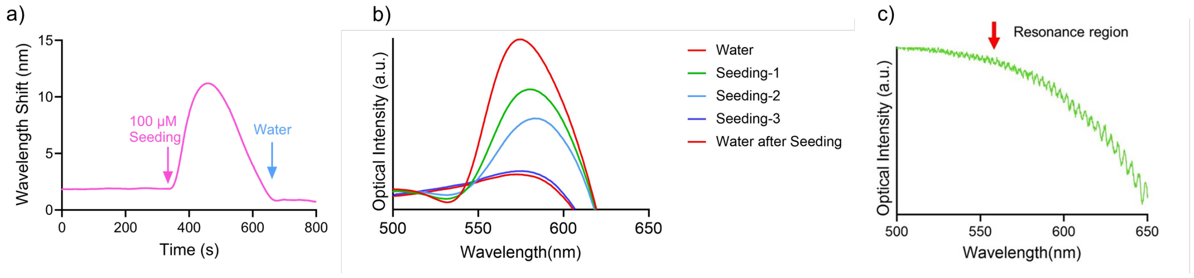

Figure S8. (a) Real-time monitoring of NI formation on a PLL (0.5 mg/mL)-modified gold-top surface. (b) Resonance shift observed upon sequential application of seeding solutions on the PLL (0.5 mg/mL)-modified gold-top surface. (c) Loss of plasmonic resonance due to excessive integration of AuNP on the sensor surface.

a)

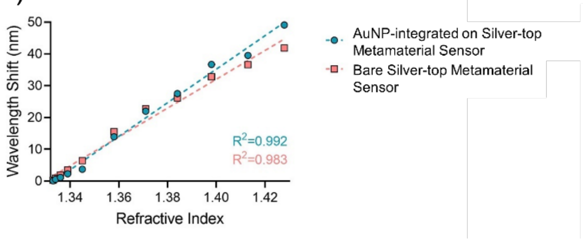

b)

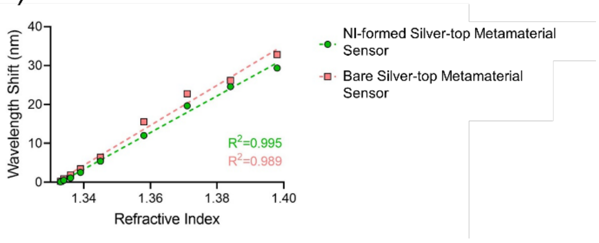

Figure S9. (a) Comparison of the bare and AuNP-integrated silver-top metamaterial sensors. (b) Comparison of the bare and NI-formed silver-top metamaterial sensors.

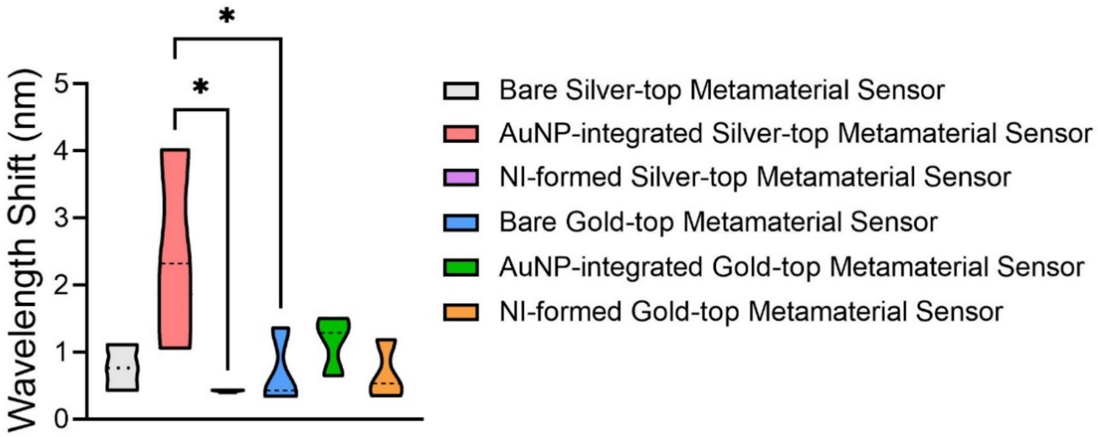

Figure S10. Violin-box plots representing sensor responses for each condition of gold-top and silver-top sensors during the detection of 10^5 particles/μL (equivalent to 1380 EVs/μL). Data were statistically analyzed using the non-parametric Kruskal–Wallis test, with significant differences indicated by an asterisk (*, $p < 0.05$; $n = 3$). Among the tested configurations, the AuNP-integrated silver-top sensor demonstrated more sensitivity than those of to the NI-formed silver-top and bare gold-top sensors.

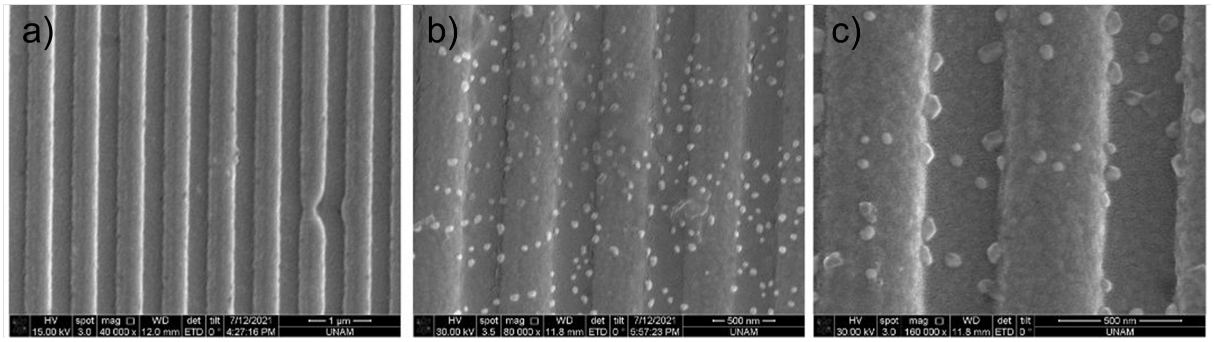

Figure S11. The SEM images of (a) the bare gold top sensor and (b-c) the captured EVs on the sensor.

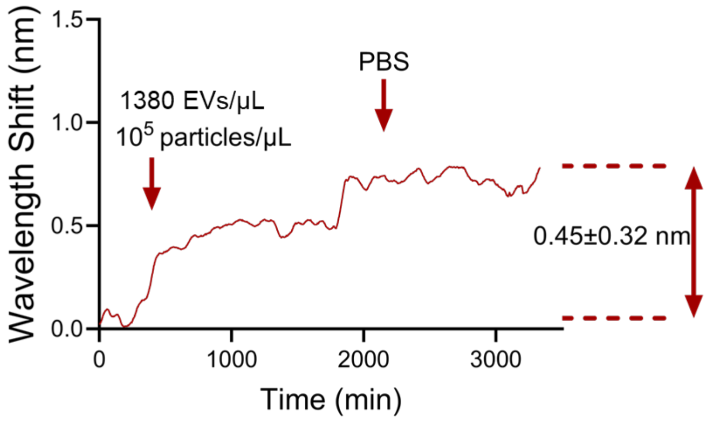

Figure S12. Detection of 10^5 particles/µL (1380 EVs/µL) on the sensor surface in the absence of anti-CD81 antibody, serving as a negative control to assess non-specific binding.

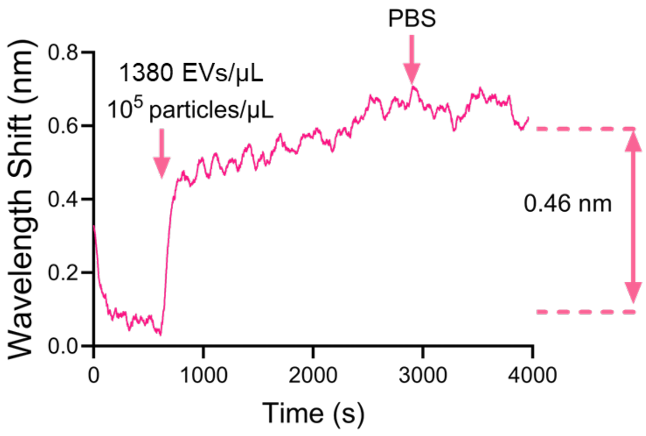

Figure S13. Detection of 10^5 particles/µL (1380 EVs/µL) on a sensor surface functionalized with anti-EpCAM antibodies, demonstrating specific binding capability toward EpCAM-positive EVs.

Table S1. Bulk sensitivity values of the sensors along with corresponding standard deviations, derived from refractive index-dependent spectral shifts.

Refractive Index (a.u.)	Gold-top metasurface (nm)	Silver-top metasurface (nm)
1.333	0.30±0.21	0.26±0.17
1.334	0.73±0.21	0.90±0.13
1.336	1.71±0.23	1.88±0.14
1.339	2.81±0.26	3.47±0.15
1.345	5.58±0.26	6.43±0.11
1.358	12.40±0.23	15.54±0.14
1.371	17.61±0.20	22.77±0.10
1.384	22.53±0.21	26.14±0.10
1.398	28.97±0.19	32.81±0.09
1.41299	34.19±0.16	36.60±0.10
1.42789	38.82±0.08	41.87±0.10

Table S2. Sensor responses for EV detection, presented with corresponding standard deviations.

Measurement Number	Gold-top metasurfaces			Silver-top metasurfaces		
	**Bare (nm)**	**AuNPs-integrated (nm)**	**NI-formed (nm)**	**Bare (nm)**	**AuNPs-integrated (nm)**	**NI-formed (nm)**
**1**	0.75±0.20	0.94±0.44	1.4±0.47	1.11±0.04	2.32±0.09	0.43±0.10
**2**	1.27±0.18	1.86±0.35	1.7±0.31	0.76±0.07	1.06±0.11	0.39±0.08
**3**	0.80±0.45	1.82±0.29	0.72±0.13	0.43±0.07	4.02±0.26	0.43±0.10

Table S3. Sensor responses for EV detection in artificial urine and plasma, presented with associated standard deviations.

Sample Type	Wavelength shift (nm)
EVs in Plasma on AuNPs-integrated gold-top	2.17±0.28
EVs in Artificial Urine AuNPs-integrated gold-top	1.30±0.33
EVs in Artificial Urine AuNPs-integrated silver-top	1.82±0.31

Table S4. Full width at half maximum (FWHM) and figure of merit (FOM) values for the sensor configurations.

Refractive Index (a.u.)	Gold-top metasurface		Silver-top metasurface	
	FWHM (nm)	FOM (RUI^{-1})	FWHM (nm)	FOM (RUI^{-1})
1.333	63.14766	6.420982187	59.76244	7.329018025
1.334	63.63158	6.372150432	60.40695	7.250821305
1.336	64.59966	6.276658422	61.05159	7.174260326
1.339	65.72947	6.168770264	62.98638	6.953884316
1.345	66.05236	6.138614881	65.26841	6.71075027
1.358	65.62524	6.17856788	68.1419	6.427763241
1.371	68.2911	5.937376906	70.3482	6.226172098
1.384	70.78871	5.727890789	59.86179	7.316854374
1.398	67.62553	5.995812528	60.94382	7.186946929
1.41299	71.04913	5.706896059	59.25233	7.392114369
1.42789	70.51586	5.750053959	58.0691	7.542737876

Table S5. Overview of various biosensing platforms reported for EV detection.

Platform	Principle	Sensitivity	Advantages	Disadvantages	Ref.
Intravesicular Nanoplasmonic System (iNPS)	Transmission	10^4 EVs in 0.5 µL sample<br><br>(2 x 10^4 EVs/µL)	• 10 × 10 of array<br>• Low sample volume (0.5 µL)	• Lengthy, complicated, and high-cost fabrication of sensors (16-26 hours)<br>• The need for labelling (AuNPs labeled antibody) for signal amplification	[1]
Plasmonic interferometer array (PIA)	Transmission	3.86×10^8 exosomes/mL	• 5 × 5 of array<br>• Smartphone integration	• The need for a microscope-based optical setup<br>• 30 times reduction in sensitivity after the smartphone integration	[2]
Nanoplasmonic Exosome (nPLEX) Sensor	Transmission	3000 EVs	• Label-free detection<br>• High throughput	• The need for a customized microscope-based optical setup	[3]
Fluorescence-amplified EV sensing technology (FLEX)	Fluorescence	2 × 10^3 EVs/µL	• Ability to detect small EVs	• Multi-step (7 fabrication step including lithography) fabrication of sensors<br>• Labelling (primary and secondary antibodies) for producing and amplifying signals<br>• The need for a customized microscope-based optical setup<br>• Multi-step image processing for analysis via image j and custum based MATLAB code (background signals correction, EV locations finding, averaged fluorescence intensities measurement etc.)	[4]
Aptamer/AuNP biosensor for colorimetric profiling	Color change (naked-eye detection)	7 µg/mL	• Label-free detection	• Semi-quantitative detection<br>• The need for a benchtop spectrometer for quantitative results	[5]
Nanobowtie-based SERS platform	SERS	1.32×10^5 particles/ml.	• Ability to distinguish EVs from liposomes	• The need for labelling cells<br>• Indirect labeling	[6]
Compact SPR	Reflectance	2×10^{10} exosomes/mL	• Detecting EVs from patient samples	• Multi-component optical setup including a prism and a mirror	[7]

Custom-made SPR platform	Reflectance	2070 exosomes/µL	• Detecting exosomes from serum samples	• Labelling with antibodies or amplifying signals	[8]
Biacore 2000 instrument for SPR measurements		4 µg/mL	• Commercially available<br>• Label-free detection	• Old version of the device (T200 new version)<br>• High cost of the instrument ($200,000–$300,000 USD)	[9]
Magnetic nanoparticle (MNP) enhanced SPR	Reflectance	0.79 µg/mL	• Reducing diffusion-limited mass transfer of analytes	• The need for sample pre-processing<br>• Labelling (MNP-labeled antibody) for signal amplification	[10]
SPR	Reflectance	$5×10^3$ particles/mL	• Better sensitivity compared to commercial ELISA<br>• Detection from complex matrices	• Labelling (two different AuNP-labeled aptamers) for signal amplification	[11]
Gold Nanoribbon enhanced SPR	Reflectance	Lipid vesicle detection 8.7–16 µg/mL	• DVD based chip fabrication	• Additional fabrication step (tape-based or PDMS mold replication)<br>• No complex specimens (e.g., serum) has not been tested<br>• Lipid vesicles do not mimic the complex structures of EVs	[12]
Optical pickup unit (OPU)	Reflectance	25 ng/mL	• Computer driver read out systems	• Nanogold or silver labelling proteins<br>• Necessity of further analysis for quantitative results and integrating a camera into the system	[13]
Spatial designs on plasmonic metamaterial sensors	Reflectance	138 EVs/µL	• ~5.5 signal enhancement without any labelling (label-free detection)<br>• Detection in complex biological specimens (artificial urine and plasma)<br>• Inexpensive sensor fabrication ($0.90 per silver-top sensor and $1.50 per gold-top sensor)—reducing the cost 260 and 154 times, respectively<br>• Short time required for fabrication—reducing the time 960 times	• To make it more universal, the platform needs to be expanded to the use of different biological matrices (*e.g.,* blood, lavage fluid, saliva, and sweat)<br>• For a clinical study in the future, the platform needs to be evaluated with patient samples, therefore addressing a clinical question specifically	This study

			• Portable setup (7.5 cm x 5 cm x 3 cm)<br>• Repurposing off-the-shelf materials		

REFERENCES

[1] J. Park, H. Im, S. Hong, C. M. Castro, R. Weissleder, and H. Lee, “Analyses of Intravesicular Exosomal Proteins Using a Nano-Plasmonic System,” *ACS Photonics*, vol. 5, no. 2, pp. 487–494, 2018, doi: 10.1021/acsphotonics.7b00992.

[2] X. Zeng *et al.*, “Plasmonic interferometer array biochip as a new mobile medical device for cancer detection,” *IEEE J. Sel. Top. Quantum Electron.*, vol. 25, no. 1, 2018, doi: 10.1109/JSTQE.2018.2865418.

[3] H. Im *et al.*, “Label-free detection and molecular profiling of exosomes with a nano-plasmonic sensor,” *Nat. Biotechnol.*, vol. 32, no. 5, pp. 490–495, 2014, doi: 10.1038/nbt.2886.

[4] M. H. Jeong *et al.*, “Plasmon-Enhanced Single Extracellular Vesicle Analysis for Cholangiocarcinoma Diagnosis,” vol. 2205148, pp. 1–12, 2023, doi: 10.1002/advs.202205148.

[5] Y. Jiang *et al.*, “Analytical Methods Aptamer / AuNP Biosensor for Colorimetric Profiling of Exosomal Proteins Angewandte,” vol. 32611, pp. 11916–11920, 2017, doi: 10.1002/anie.201703807.

[6] M. Jalali *et al.*, “Plasmonic nanobowtiefluidic device for sensitive detection of glioma extracellular vesicles by Raman spectrometry,” *Lab Chip*, vol. 21, no. 5, pp. 855–866, 2021, doi: 10.1039/d0lc00957a.

[7] C. Liu *et al.*, “Sensitive Detection of Exosomal Proteins via a Compact Surface Plasmon Resonance Biosensor for Cancer Diagnosis,” *ACS Sensors*, vol. 3, no. 8, pp. 1471–1479, 2018, doi: 10.1021/acssensors.8b00230.

[8] A. A. I. Sina, R. Vaidyanathan, S. Dey, L. G. Carrascosa, M. J. A. Shiddiky, and M. Trau, “Real time and label free profiling of clinically relevant exosomes,” *Sci. Rep.*, vol. 6, no. 1, pp. 1–9, 2016, doi: 10.1038/srep30460.

[9] D. L. M. Rupert *et al.*, “Determination of exosome concentration in solution using surface plasmon resonance spectroscopy,” *Anal. Chem.*, vol. 86, no. 12, pp. 5929–5936, 2014, doi: 10.1021/ac500931f.

[10] A. T. Reiner, N. G. Ferrer, P. Venugopalan, R. C. Lai, S. K. Lim, and J. Dostálek, “Magnetic nanoparticle-enhanced surface plasmon resonance biosensor for extracellular vesicle analysis,” *Analyst*, vol. 142, no. 20, pp. 3913–3921, 2017, doi: 10.1039/c7an00469a.

[11] Q. Wang *et al.*, “Direct quantification of cancerous exosomes via surface plasmon resonance with dual gold nanoparticle-assisted signal amplification,” *Biosens. Bioelectron.*, vol. 135, no. October 2018, pp. 129–136, 2019, doi: 10.1016/j.bios.2019.04.013.

[12] C. Zhao *et al.*, “Scalable Fabrication of Quasi-One-Dimensional Gold Nanoribbons for Plasmonic Sensing,” *Nano Lett.*, vol. 20, no. 3, pp. 1747–1754, 2020, doi: 10.1021/acs.nanolett.9b04963.

[13] H. Z. Yu, Y. Li, and L. M. L. Ou, “Reading disc-based bioassays with standard computer drives,” *Acc. Chem. Res.*, vol. 46, no. 2, pp. 258–268, 2013, doi: 10.1021/ar300104b.
